# Shifts in the microbiome and virome are associated with stony coral tissue loss disease (SCTLD)

**DOI:** 10.1093/ismeco/ycaf226

**Published:** 2025-11-27

**Authors:** Shrinivas Nandi, Timothy G Stephens, Kasey Walsh, Rebecca García-Camps, Maria F Villalpando, Rita I Sellares-Blasco, Ainhoa L Zubillaga, Aldo Croquer, Debashish Bhattacharya

**Affiliations:** Microbial Biology Graduate Program, Rutgers University, 76 Lipman Drive, New Brunswick, NJ, 08901, United States; Department of Biochemistry and Microbiology, 59 Dudley Road, Rutgers University, New Brunswick, NJ, 08901, United States; Microbial Biology Graduate Program, Rutgers University, 76 Lipman Drive, New Brunswick, NJ, 08901, United States; Fundación Puntacana, Marine Innovation Centre, Puntacana Resort & Club, Punta Cana 23000; Fundación Dominicana de Estudios Marinos, Marine Research Dominican Republic, Calle Los Manantiales, Bayahibe, La Altagracia, 23000, Dominican Republic; Fundación Dominicana de Estudios Marinos, Marine Research Dominican Republic, Calle Los Manantiales, Bayahibe, La Altagracia, 23000, Dominican Republic; Fundación Puntacana, Marine Innovation Centre, Puntacana Resort & Club, Punta Cana 23000; The Nature Conservancy, Caribbean Division, Central Caribbean Program, Avenida 27 de Febrero, Plaza Central 1, Santo Domingo, 10148, Dominican Republic; Department of Biochemistry and Microbiology, 59 Dudley Road, Rutgers University, New Brunswick, NJ, 08901, United States

**Keywords:** metagenomics, coral disease, viral genomics, microbiome

## Abstract

Stony coral tissue loss disease (SCTLD) is a rapidly spreading lethal coral disease, the etiology of which remains poorly understood. In this study, using deep metagenomic sequencing, we investigated microbial and viral community dynamics associated with SCTLD progression in the Caribbean stony coral *Diploria labyrinthiformis*. We assembled 264 metagenome-assembled genomes and correlated their abundance with disease phenotypes, which revealed significant shifts in both the prokaryotic microbiome and virome. Our results provide clear evidence of microbial destabilization in diseased corals, suggesting that microbial dysbiosis is an outcome of SCTLD progression. We identified DNA viruses in our dataset that increase in abundance in SCTLD-affected corals and are present in existing coral data from other Caribbean regions. In addition, we identified the first putative instance of asymptomatic/resistant SCTLD-affected corals. These are apparently healthy colonies that share the viral profile of diseased individuals. However, these colonies contain a different prokaryotic microbiome than do diseased corals, suggesting microbe-induced resilience (i.e. beneficial microbiome) to SCTLD. Finally, utilizing differential abundance analysis and gene inventories, we propose a mechanistic model of SCTLD progression, in which viral dynamics may contribute to microbiome collapse. These findings provide novel insights into SCTLD pathogenesis and offer consistent molecular signals of disease across diverse geographic sites, presenting new opportunities for disease monitoring and mitigation.

## Introduction

In the past decade, coral reefs in the Caribbean have emerged as a hotspot of coral diseases [1, 2]. Stony coral tissue loss disease (SCTLD) was first observed in 2014 in Miami-Dade County and the Florida Keys [3]. Sediment resuspension caused by a dredging operation in Miami Harbor is believed to have contributed to the emergence of this disease [4], however the cause remains unknown. SCTLD affects more than 22 different coral species with varying levels of susceptibility [5]. Evidence of active SCTLD infection includes focal or multifocal lesions that spread at chronic to acute rates, followed by tissue loss [3, 5]. The SCTLD epidemic has decimated affected reefs, with a fatality rate of 73%–100% [6] and is transmissible through both direct contact and seawater, even from the ballast of ships [4, 7–11]. Since 2014, SCTLD has spread throughout coral reefs in Florida and is now present in Mexico, Turks and Caicos, the U.S. Virgin Islands, the Dominican Republic, and many other sites across the Caribbean [12–15].

No SCTLD-associated causative agent is known that satisfies Koch’s postulates [16]. Elucidating the cause of coral diseases has been a challenge, wherein of 18 known coral diseases, Koch’s postulates have been satisfied for only five [17]. A vast proportion of initial SCTLD studies focused on utilizing 16S rRNA sequencing to identify causative prokaryotes [5]. These studies identified many orders of bacteria that are strongly correlated with infection, including Flavobacteriales*,* Clostridiales*,* Rhodobacterales*,* Alteromonadales*,* Vibrionales*,* Rhizobiales, and Campylobacterales [5, 18–21]. These findings, paired with an alleviation of the disease upon treatment with antibiotics [22–24] and probiotics [25, 26] initially suggested a bacterial causative agent. However, a single causative bacterial pathogen present in all SCTLD-affected coral species has not yet been identified; instead, the data suggest the involvement of a polymicrobial consortium [27, 28].

Whereas 16S rRNA-based studies have provided novel insights, they have largely excluded other potential causative agents, such as viruses and eukaryotes. For example, one study identified an increased abundance of the micro-eukaryote *Cillaphora* in SCTLD-affected corals [28]. Viral-like particles (VLPs) have also been observed using microscopy within the algal endosymbionts of SCTLD-affected corals [29], which, when paired with metatranscriptomics, led to the hypothesis that a viral agent may induce endosymbiont dysfunction and autophagy [30]. RNA viruses, specifically *coral-associated alphaflexiviruses*, have also been linked to diseased corals [31]. A few studies have assessed the role of DNA viruses in SCTLD, however only a handful of viral contigs were identified in association with the disease phenotype [32]. However, other studies have reported the presence of filamentous VLPs in endosymbionts from regions where SCTLD is not present [33], raising questions about their role in the disease. In addition, histopathological studies have shown discrepancies in lesion severity and cellular involvement, suggesting regional differences and variable disease presentations [34].

In this study, we analyzed the holobiont metagenome of *Diploria labyrinthiformis*, a SCTLD-susceptible coral present in reefs of the Dominican Republic [16]. Samples were collected from affected and healthy colonies (HC) near and far away from the disease lesion and analyzed using Illumina metagenomic sequencing, followed by bioinformatic analysis. These results were used to derive a putative model of SCTLD progression, in which a viral infection of the holobiont is hypothesized to cause dysbiosis of the microbiome, making the coral more vulnerable to a secondary infection by opportunistic pathogens. We propose that these biotic shifts result in the visual symptoms of SCTLD.

## Materials and methods

### Field sampling

**Figure 1:**
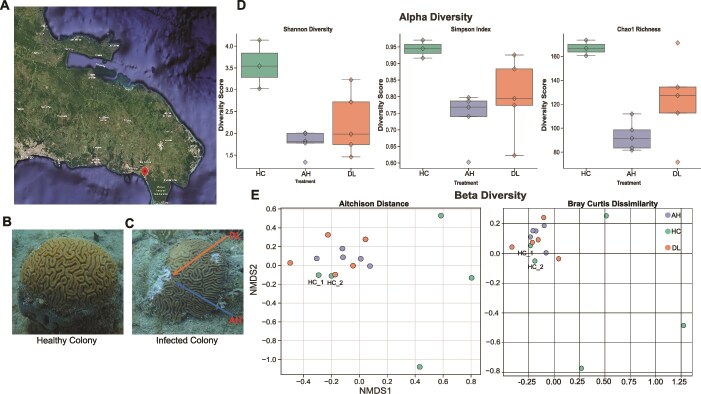
**Experimental design and microbiome ecological diversity parameters. (A)** Satellite image showing the coordinates of the sample collection site in Playita reef (18.373081, −68.853475), Dominican Republic. Image taken from Google earth. **(B)** Image of a healthy *D. Labyrinthiformis* colony, i.e. no signs of SCTLD. Scrapings shown are those caused during sample collection. **(C)** Image of a SCTLD affected *D. Labyrinthiformis* colony. The lower arrow indicates where the AH samples were collected, and the upper arrow shows where the DL samples were collected. **(D)** Alpha diversity metrics (Shannon diversity, Simpson index, and Chao1 richness) for all MAGs from the three sample types (healthy Colony [HC]: left, apparently healthy [AH]: middle, and diseased lesion [DL]: right). The asymptomatic colonies N31 and N37 were removed from the analysis for this plot. **(E)** Beta diversity shown as NMDS plots generated from Aitchison distance (stress score 0.128) and bray Curtis dissimilarity (stress score 0.072) metrics. A clear clustering of “healthy” samples HC_1 and HC_2 (labeled) with affected colonies is observed.

Samples of the coral *D. labyrinthiformis* were collected from Playita Reef (18.373081–68.853475), Bayahibe, Dominican Republic on 12 June 2024 (Fig. 1A). The samples were collected from an average depth of 5.82 m using SCUBA. All sampled colonies were approximately 5 m apart from one another, with the overall collection area spanning ~250m^2^.

Each colony represents a single replicate in our study. In total, five HC, showing no visible signs of SCTLD, were collected (Fig. 1B). In addition, we identified five colonies that displayed macroscopic signs of SCTLD [35]: i.e. were diseased.

From each diseased colony (DC) we sampled two distinct tissue types: one directly from the active diseased lesion (hereinafter “DL”) and one approximately 2 inches away from the lesion in an area of tissue that appeared visually unaffected (hereinafter “AH”) (Fig. 1C). The ~2-inch distance from the active lesion was chosen to: (i) minimize contamination from necrotic tissue directly at the lesion edge while, (ii) capturing the transition zone between diseased and apparently healthy (AH) tissue where microbial interactions relevant to disease progression are most likely to occur. All samples were collected using a hammer and chisel and immediately stored in individual Whirl-Pak bags to prevent cross-contamination.

After collection, samples were stored in a cooler filled with seawater and transported to shore for processing. Approximately 0.25 g of each sample was placed into 2 mL tubes containing 2 mm silica beads and 1 mL of Zymo DNA/RNA Shield. Samples were vortex-homogenized in the presence of silica beads for 10 minutes, until all tissue was stripped off the coral skeleton. Samples were then transported by air to Rutgers University within 2 days of collection. Upon arrival, samples were stored at −80°C until further processing.

Ideally, a more extensive sampling of different colony genotypes exhibiting macroscopic signs of SCTLD-related health states would have been done to strengthen the results described in this study. However, the dire situation faced by reefs in the Dominican Republic, with low population sizes for many species, led us to reduce our effort to minimize damage to surviving, wild corals. Our overarching goal was to identify potential biomarkers of SCTLD for future analyses that could be tested more broadly using non-destructive sampling approaches, such as analysis of coral mucus.

### DNA extraction and sequencing

Previous coral microbiome studies have focused on microbe-rich mucus excreted by the host. However, this approach may fail to enrich for intracellular microbes and viruses, which are one of the hypothesized infectious agents in SCTLD [29, 32]. We therefore homogenized and extracted DNA from the collected coral samples (host and mucus combined) using the ZymoBIOMICS DNA extraction kit, based on the protocol provided by the manufacturer, with the average yield being 5321.9 ± 2562.4 ng of total DNA. Samples were sent in two batches for sequencing. The first six samples were sent to assess the extraction procedure, and once validated, the remainder were sent for processing. Sequencing was done by Azenta Life Sciences using paired-end (2x150 bp) Illumina shotgun metagenomic sequencing reagents on a NovaSeq X+, targeting 90 Gbp of read data for each sample.

### Construction of metagenome assembled genomes

Metagenome Assembled Genomes (MAGs) were constructed for prokaryotes, eukaryotes, plasmids, and viruses using a custom workflow termed Naïve ATLAS (https://github.com/TimothyStephens/naive_atlas). This workflow combined the highly automated ATLAS workflow (https://github.com/metagenome-atlas/atlas; only designed for prokaryotes) [36] with the taxonomically agnostic (but less automated) VEBA v2 (https://github.com/jolespin/veba) workflow [37].

Raw metagenomic reads were deduplicated, trimmed, error corrected, and removed if they were putatively from the draft *D. labyrinthiformis* genome (see below) using the bbmap v39.13 package (https://sourceforge.net/projects/bbmap) tools clumpify.sh (dedupe=t dupesubs=2 optical=false), bbduk.sh (qout=33 trd=t hdist=1 k=27 ktrim=r mink=8 trimq=10 qtrim=rl minlength=51 maxns=−1 minbasefrequency=0.05 ecco=t prealloc=t), tadpole.sh (mode=correct aggressive=false tossjunk=f tossdepth=1 merge=t shave=f rinse=f), and bbsplit.sh (maxindel=20 minratio=0.65 minhits=1 ambiguous=best k=13 local=t machineout=t), respectively. The cleaned read pairs were merged using bbmerge.sh (k = 62), before being assembled by megahit v1.2 (−-k-min 21 --k-max 121 --k-step 20 --min-contig-len 1000 --min-count 2 --merge-level 20,0.98 --prune-level 2 --low-local-ratio 0.2) [38]. The cleaned reads from each sample were mapped against each megahit assembly using minimap2 v1.19.0 (−x sr) [39], with contig abundance calculated using the jgi_summarize_bam_contig_depths (−-percentIdentity 95) tool from metabat2 v2.15 [40]. MAG construction was performed in three sequential stages, described below.

Stage 1: Prokaryotic MAGs were identified and extracted from each assembly first. Initially, bins were constructed using the contig abundance information and raw assemblies by MetaBAT2 v2.15 (−m 1500 --minClsSize 150 000 --seed 1), MaxBin v2.2.7 (−min_contig_length 1500 -markerset 107) [41], and MaxBin again using the alternative marker set (−min_contig_length 1500 -markerset 40). DAS_Tool v1.1.2 (−-search_engine diamond --score_threshold 0.1 --write_bins 1 --create_plots 1) [42] was used to combine the different bins into a unified non-redundant set. Non-prokaryotic contigs were removed from the DAS tools bins using whokaryote.py v1.1.2 (−-minsize 1500) [43] and mdmcleaner v0.8.7 (using the “clean” command) [44]. CheckM2 v1.0.2 (using the “predict” command) [45, 46] was used to assess the integrity of the cleaned bins, keeping only those with completeness >50% and contamination <10%. The cleaned and filtered bins from each sample were clustered into a final non-redundant set using skani v0.1 (triangle --sparse --ci --min-af 20) [47] and the representative selection script provided by VEBA (pre-cluster threshold 92.5% and ANI threshold 95%). Taxonomy was assigned using GTDB-Tk v2.4 (using the “identify,” “align,” and “classify” commands; default parameters) [48]. The bins produced by this process constitute the final prokaryotic MAGs (pMAGs) used for downstream analysis.

Stage 2: Next, the contigs not binned in the previous step (i.e. non-prokaryotic) were assessed and used to construct eukaryotic MAGs. Bins were constructed using MetaBAT2 v2.15 (−m 1500 --minClsSize 2000000 --seed 1), with putative non-eukaryotic contigs removed from each bin using whokaryote.py v1.1.2 (−-minsize 1500). BUSCO v5.4 (−m genome --auto-lineage-euk --evalue 0.001) [49] was used to assign taxonomy and completeness to each bin; only bins with completeness >10% were retained for downstream analysis. A low completeness cutoff was used due to the challenges associated with binning eukaryotic MAGs. It is difficult to assemble and assess the completeness of short read-based eukaryotic genomes, thus a lower threshold is required, if only to gain a view of which taxa are present across the samples. The eukaryotic bins from each sample were combined into a non-redundant set (95%) of MAGs (eMAGs) using the same approach and cutoffs as for the prokaryotes (skani and the custom VEBA script).

Stage 3: Finally, the contigs not binned in the previous steps (i.e. non-prokaryotic and non-eukaryotic) were used to construct viral and plasmid MAGs. Bins were constructed using MetaBAT2 v2.15 (−m 1500 --minClsSize 2500 --seed 1), with bin completeness and taxonomy assessed using geNomad v1.9.0 (end-to-end --cleanup --verbose --enable-score-calibration --disable-find-proviruses --sensitivity 4.0 --splits 0 --composition auto --min-score 0.7 --max-fdr 1.0 --min-plasmid-marker-enrichment −100 --min-virus-marker-enrichment −100 --min-plasmid-hallmarks 0 --min-virus-hallmarks 0 --max-uscg 100) [50]. Only bins with a False Discovery Rate < 0.05 were retained. Bins from each sample were combined (viruses and plasmids separately) into a non-redundant set (95%) of MAGs (vMAGs and pMAGs, respectively) using the same approach and cutoffs as for the prokaryotes and eukaryotes (skani and the custom VEBA script).

Gene prediction was done separately for each type of non-redundant MAG. Of the pMAGs, those taxonomically assigned as bacteria had genes predicted using bakta v1.10.4 (−-meta --keep-contig-headers) [51], with those assigned as archaea predicted using prokka v1.14.6 (−-addgenes --addmrna --metagenome --kingdom Archaea) [52]. Genes were predicted in eMAGs using the “eukaryotic_gene_modeling_wrapper.py” script from VEBA (using the VEBA custom MicroEuk50 database; −-metaeuk_sensitivity 4.0 --metaeuk_evalue 0.01 --pyrodigal_minimum_gene_length 90 --pyrodigal_minimum_edge_gene_length 60 --pyrodigal_maximum_gene_overlap_length 60 --pyrodigal_mitochondrial_genetic_code 4 --pyrodigal_plastid_genetic_code 11 --barrnap_length_cutoff 0.8 --barrnap_reject 0.25 --barrnap_evalue 1e-06 --trnascan_mitochondrial_searchmode = “-O” --trnascan_plastid_searchmode = “-O”), in vMAGs using prokka v1.14.6 (−-addgenes --addmrna --metagenome --kingdom Viruses), and in plMAGs using bakta v1.10.4 (−-meta --keep-contig-headers).

To aid MAG construction and reduce overall computational load, a preliminary short-read-only *D. labrynthiformis* reference genome was constructed using the first round of sequencing data. Naïve ATLAS was utilized to clean the read data and assemble contigs. BLASTN (v.2.13.0) was performed against the nt database (2022_07), to assess taxonomy. Blobtools (v 1.1) [53] was used to visualize the taxonomy and subsequently to filter for cnidarian contigs. The resulting cnidarian contigs were filtered for viruses using geNOMAD. The final cleaned cnidarian contigs were used as an internal reference for Naïve ATLAS. All downstream analyses were performed using Python v3.9.7 with the base packages, pandas (v2.2.3), and numpy (v1.26.4). Plots were generated using matplotlib (v3.6.2) and seaborn (v0.12.1). Statistical tests were performed using SciPy (v1.13.1), unless specified otherwise.

#### Assessing the algal endosymbiont population

A database of endosymbionts nuclear genomes from seven genera [54–61] was constructed and indexed using Bowtie2 (v2.5.4) [62]. Cleaned metagenomic reads were mapped to this combined database using bowtie2 (−-end-to-end), with CoverM (−-genome, v.0.6.1) [63] utilized to generate TPM counts for the algal endosymbionts. Relative abundances were calculated for the endosymbionts using TPM values.

### Microbiome and virome: species diversity, relative abundance, and differential abundance analysis

Alpha diversity was assessed using Shannon diversity, Simpson evenness index, and Chao1 richness (scikit-bio v0.6.3 using function alpha) metrics. A Shapiro–Wilk test was utilized to assess normality of the data. Due to the data not being normally distributed, the non-parametric Kruskal-Wallis test was done to assess significant shifts between health conditions. Furthermore, the Mann–Whitney U test was used to assess pairwise community shifts in the three health conditions, with a Benjamini–Hochberg correction used for multiple testing. Beta diversity among samples was assessed using both Aitchison distance and Bray–Curtis dissimilarity [64]. For Aitchison distance, a centred log-ratio (CLR) transformation was applied to the data, followed by the generation of a dissimilarity matrix (scipy.spatial.distance: pdist, squareform, and skbio.stats.composition: clr) [65]. To further assess the sample clustering profile, we used the same dissimilarity matrix in a non-metric multidimensional scaling (NMDS) analysis using sklearn.manifold MDS (scikit-learn v1.6.1). We performed a PERMANOVA analysis (999 permutations) to test whether community centroids differed between health states (i.e. whether overall community composition varied). To account for differences in within-group variability, we also conducted a PERMDISP analysis, which assessed whether observed differences could be attributed to variation in dispersion (i.e. community variability [a proxy of beta diversity]). Using both tests allowed us to distinguish true compositional shifts among health states from differences driven by heterogeneous dispersion.

Relative abundance was calculated using TPM values generated separately for prokaryotes (pMAGs) and viruses (vMAGs) in each sample to prevent shifts in one affecting the apparent relative abundance of MAGs in the other. This allowed independent assessment of the prokaryotic microbiome (hereinafter *microbiome*) and the viral microbiome (hereinafter *virome*). Microbiome shifts were evaluated at the class level, whereas virome shifts were assessed at the phylum level (based on the Baltimore classification).

Numerous tools exist to assess differential abundance in metagenomic data. The non-normally distributed data, paired with its heterogenous nature and the large number of zero values made it challenging to select a single best approach [66]. Conservative tools such as ANCOM-BC [67] and Aldex2 [68] have been championed as the most reliable for microbiome analyses. However, these tools often require many samples per group, a minimum of *n* = 10 and *n* = 8 (respectively), whereas our study only had *n* = 5. Furthermore, studies have also assessed the utilization of tools designed for RNA-seq data analysis, such as edgeR [69] and limma-voom, however these are not recommended due to high false positive rates [70, 71]. Another such tool recommended for metagenomic analysis is DESeq2 (or pyDESeq2 v0.4.8) [72], which can run with fewer samples per group (*n* = 3).

MAGs were classified as SCTLD-associated if they were differentially abundant (|log₂FC| > 2.0 and adjusted *P*-value <.05) in AH and DL compared to HC. In addiiton, MAGs were biologically relevant (i.e. of sufficient abundance to have a significant effect on the overall microbiome) if they had a median relative abundance >10% in either healthy or diseased colonies. Using these thresholds, a set of pMAGs and vMAGs were selected that showed a significant shift in diseased colonies (AH and DL) when compared to HC. TPM based box plots were generated for each selected MAG to visualize and investigate the apparent abundance shifts.

### Verifying vMAGs and putative host identification

To confirm that all scaffolds assigned to each of the selected vMAGs are of viral provenance, and to identify their putative hosts, a phylogenetic approach was applied to each protein predicted in each vMAG. This analysis used the nr (2022_07) and IMG/VR (v4.1) databases and the DIAMOND blastp (v2.1.10.164; −-ultra-sensitive --max-target-seqs 0 --evalue 1e-05) [73], MAFFT (v7.520; −-auto) [74], trimAl (v1.5.rev0; −-automated1) [75] and iqtree (v2.3.6; −m TEST -bb 1000) programs. A detailed description of the methods is presented in the Supplemental Text 1.

### Downstream assessment of prokaryotic MAGs

The taxonomy and quality of pMAGs was assessed using the Naïve ATLAS workflow (described above). Functional annotation of proteins from selected pMAGs was performed using GhostKOALA [76]. Furthermore, virulence capabilities of these pMAGs were assessed using a DIAMOND blastp search against the virulence factor database (VFDB 2022) [77].

### Assessing other SCTLD metagenomic studies

To test the association of our selected MAGs with SCTLD, we assessed their presence in the only other SCTLD metagenomic dataset available, NCBI BioProject PRJNA576217 [28]. These authors generated 58 short-read metagenomic datasets from four coral species in Florida: *D. labyrinthiformis*, *Stephanocoenia intersepta*, *Meandrina meandrites*, and *Dichocoenia stokesii*. These samples were annotated as DL, Disease Unaffected (“DU,” hereinafter “AH”) and lastly, Apparently Healthy (“AH,” hereinafter “HC”). Briefly, reads were mapped against all our MAGs using bowtie2 (−-local) (v2.5.4) [62], with coverage calculated using CoverM (−-genome) (v.0.6.1) [63]. A MAG was present in a species if it had a TPM > 1.0 in at least 5 samples (out of ~15 samples per species). See Supplemental Text 1 for a full description.

## Results

### General metagenome analysis

A detailed description of the sequencing and MAG construction results is presented in the Supplemental Text 1. Briefly, a total of 264 MAGs were identified, two were eukaryotic (although highly incomplete and not used for downstream analysis), 85 were prokaryotic MAGs (pMAGs; Supplemental Table 1), 36 were plasmid MAGs, and 141 were viral MAGs (vMAGs; Supplemental Table 2). Of the 141 vMAGs, 109 were classified as Caudoviricetes, which are widely distributed bacteriophages.

### Algal endosymbiont composition

In the HC samples we observed a mix of *Durusdinium trenchii* (0.21 ± 0.20) and *Breviolum minutum* (0.57 ± 0.46). One sample also had the endosymbiont *Cladocopium goreaui* at a relative abundance of 0.06 [Supplemental Fig. 1, Supplemental Table 3]. One AH sample had no reads mapped to any of the endosymbiont genomes, however, in the remaining AH samples, *Symbiodinium necroappetens* (0.44 ± 0.28) was the most abundant, followed by *D. trenchii* (0.20 ± 0.17) and *B. minutum* (0.00 ± 0.06). Lastly in the DL samples, *D. trenchii* (0.33 ± 0.18) was the most abundant, followed by *S. necroappetens* (0.11 ± 0.37), and *B. minutum* (0.05 ± 0.09).

### Microbial species metrics

In all three indices used in this study, HC had the highest diversity and richness (0.944 ± 0.027, 3.568 ± 0.557, and 166.917 ± 6.502, respectively), followed by DL (0.8 ± 0.117, 2.227 ± 0.729, and 123.431 ± 36.127), and lastly, AH (0.739 ± 0.079, 1.79 ± 0.272, and 93.46 ± 12.37) (Supplemental Table 4, Fig. 1D). Kruskal–Wallis tests revealed differences in the Simpson (adjusted *P*-value = .054), Chao1 (adjusted *P*-value = .054), and Shannon index (adjusted *P*-value = .054). Pairwise comparisons using the Mann–Whitney U test and subsequent BH corrections showed no statistical difference in the three diversity metrics.

NMDS plots (Fig. 1E) of the MAG beta diversity between each sample showed that the diseased colonies (AH and DL) clustered tightly together irrespective of genotype, suggesting they had similar microbiome and virome profiles. Of the HC, three were distributed along both axes and did not group with other samples, suggesting they had diverse and unique microbiome compositions. Two of the HC, which had no visible lesions at the time of collection (HC_1 and HC_2 in Fig. 1E), clustered tightly with the diseased colonies. Furthermore, upon reinspection of these two “healthy” colonies in the field, nine months after initial sampling, both showed no visible signs of SCTLD (Supplemental Fig. 2). Therefore, HC_1 and HC_2 were removed from subsequent analyses, unless otherwise specified. PERMANOVA was used to evaluate the significance of microbial beta-diversity across the three sample groups: i.e. HC, AH, and DL (Aitchison distance: pseudo-F = 1.63, *n* = 13, *P*-value = .008, permutations = 999; Bray Curtis Dissimilarity: pseudo-F = 3.22, *n* = 13, *P*-value = .003, permutations = 999). To confirm these findings, PERMDISP was also calculated (Aitchison distance: F-value = 13.57, *n* = 13, *P*-value = .005, permutations = 999, Bray Curtis dissimilarity: F-value = 12.36, *n* = 13, *P*-value = .013, permutations = 999). These results imply that the dispersion of samples was driving the PERMANOVA results, which may be attributed to microbial taxon variation in the healthy microbiome [Fig. 1E].

### Microbiome profile shifts under infection

**Figure 2:**
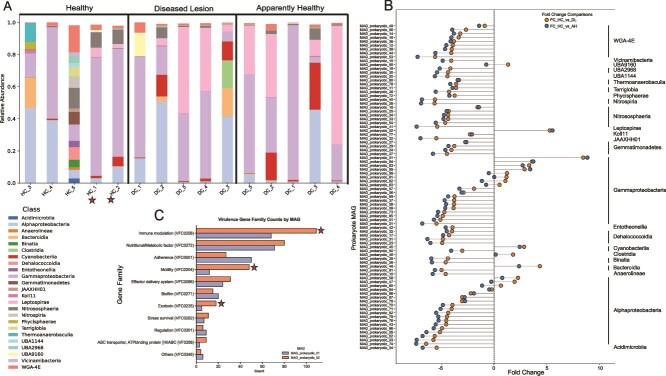
**Shifts in the prokaryotic component of the microbiome. (A)** Relative abundance (*Y*-axis) at the class level of the prokaryotic microbiome plotted as a stacked bar graph. The *X*-axis shows all samples, with the respective colony number, either HC or DC. The graph is divided into three parts, headers provided on the top, each representing the health status, i.e. HC, AH, or DL. The stars show the two putative asymptomatic colonies in this study. A legend for each class is shown on the bottom left of the image. Some samples may not add up to 1.0 because the sample has some pMAGs that do not have class level taxonomic classification. **(B)** Lollipop plot showing the log_2_FC (*X*-axis) for each prokaryotic MAG (*Y*-axis). MAGs are grouped by taxonomic class, shown on the right of the panel. The points represent the change in abundance between the HC and DL samples and the change in abundance between the HC and AH samples. **(C)** Gene abundance plot of virulence factor genes from the two pMAGs of interest: Leptospirae and Gammaproteobacteria. For each gene family (*Y*-axis) the respective VFDB ID is shown, with the number of genes identified in those families in each pMAG shown on the *X*-axis. The stars show key virulence pathways, wherein pMAG02 has more genes than pMAG01.

Class-level relative abundance analysis of the microbiome identified Alphaproteobacteria and Gammaproteobacteria as highly prevalent in HC (Supplemental Table 1; Fig. 2A; see Supplemental Text 1). Differential abundance analysis of the 85 pMAGs using DESeq2 (|log₂FC| > 2.0 and an adjusted *P*-value <.05) identified three which significantly increased in diseased colonies (AH and DL tissue). Eight pMAGs showed significantly decreased abundance in DL tissue, and seven in AH tissue, with the latter representing a subset of the former. Fold change analysis (not statistically significant) identified 16 pMAGs with increased abundance and 69 pMAGs with decreased abundance in affected colonies (Fig. 2B). pMAGs that were differentially abundant and had a relative abundance >0.1 in either AH or DL tissue were assessed further. As a result, MAG_prokaryotic_02 (hereinafter pMAG02) and MAG_prokaryotic_01 (hereinafter pMAG01) were selected for downstream analysis.

### pMAGs involved in disease phenotype

pMAG02 had a CheckM completeness of 96.82% and a contamination of 1.05%, and pMAG01 had a completeness of 72.68% and a contamination of 0.54%. At the class level pMAG02 was annotated as Leptospirae (order: Leptospirales)*.* pMAG01 was annotated as a Gammaproteobacteria, with a genus-level annotation of JANQNX01 (an uncultured Gammaproteobacterium, previously identified in Australia).

We assessed the virulence proteins in each of these pMAGs using VFDB. A total of 393 proteins were identified as virulence factors in pMAG02 and 305 proteins in pMAG01 (Supplemental Table 5). Both MAGs had similar nutrient/metabolic factors (VFC0272) at 80 and 71 (Fig. 2C), respectively. pMAG02 contained 109 immune modulation genes (VFC0258), whereas pMAG01 contained 69. Furthermore, pMAG02 had more motility (48) genes (VFC0204) compared to pMAG01 (12). Lastly pMAG02 also contained more (18) exotoxin genes (VFC0235), compared to pMAG01 (12) (red stars in Fig. 2C). pMAG02 contained key exotoxins that have been previously associated with virulence in other species, such as hemolysins *tlyA* and *tlyC*. We assessed prokaryotes that have been previously associated with SCTLD, namely Rhodobacterales, Rhizobiales*,* and *Vibrio* spp. and identified three pMAGs annotated as Rhodobacterales which had a median relative abundance in the order of 1e^−05^. No pMAGs were annotated as Rhizobiales or *Vibrio* spp.

### Virome profile shifts under infection

An initial relative abundance analysis of the virome was performed at the “phylum” level. Of the 141 viral MAGs identified in our dataset, 109 were classified as Caudoviricetes (phylum Uroviricota) (Supplemental Table 2). Seven other phyla were identified in our samples: Duplodnaviricota, Lenarviricota, Nucleocytoviricota, Peploviricota, Phixviricota, Pisuviricota, and Preplasmiviricota (for in-depth phylum level analyses see Supplemental Text 1, Supplemental Fig. 3).

**Figure 3:**
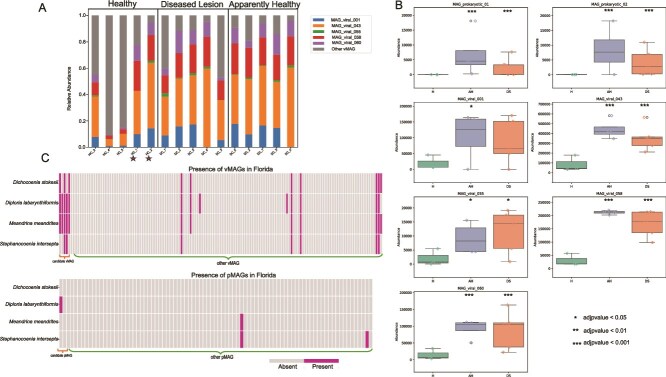
**Viral compositional analysis and MAGs correlated with SCTLD. (A)** Relative abundance (*Y*-axis) of the SCTLD-associated vMAGs and all other vMAGs. The *X*-axis represents all samples, with the respective colony number, either HC or DC. The graph is divided into three parts, headers provided on the top, each representing the health status, i.e. healthy (HC), AH, or DL. The stars show the two putative asymptomatic colonies in this study. **(B)** Box plots showing the TPM (*Y*-axis) of the MAGs of interest, across the different health conditions (*X*-axis). For this analysis, the putatively asymptomatic colonies were removed from the healthy condition (green); all five samples were used for the AH and DL conditions. Stars on the top of each box plot show the statistical significance generated by DESeq2, compared to the healthy condition. A legend is presented on the bottom right of the panel. **(C)** Presence/absence heatmap of vMAGs and pMAGs that were generated in this study in SCTLD samples from Florida, USA (see text for details).

Differential abundance analysis of vMAGs identified 17 with significantly increased abundance in AH (compared to HC) and 16 in DL, with the latter being a subset of the former (see Supplemental Text 1, Supplemental Fig. 4). To assess the biological relevance of the differentially abundant vMAGs, we examined their relative abundances in colonies with different tissue types. Among the 17 vMAGs with increased abundance in diseased colonies, only five contributed substantially to the overall virome composition based on total median relative abundance, accounting for 91.3% in AH tissue and 76.5% in DL tissue (Fig. 3A, Supplemental Table 2). These five dominant vMAGs were MAG_viral_001*,* MAG_viral_043*,* MAG_viral_055*,* MAG_viral_058*,* and MAG_viral_060 (hereafter known as SCTLD-associated vMAGs)*.* In HC, the median abundances of these five vMAGs had a total median abundance of 15.2% of the virome. None of the other 12 vMAGs that had an increased abundance in AH or DL or had a relative abundance greater than 0.1 and were therefore not considered for downstream analyses.

### vMAGs involved in the disease phenotype

In the five SCTLD-associated vMAGs, 41 proteins were predicted with only four having functional annotations predicted using GhostKOALA. Three of the proteins are from vMAG055 and were annotated as dcm (K26510), a DNA methyltransferase ERCC 2 (K10844), and a kinesin family member 21 protein KIF21 (K24185). One protein was from vMAG060 and was annotated as an adiponectin receptor ADIPOR. Of the 41 proteins from these five vMAGs, 25 (from four vMAGs [vMAG058 had no hits]) had hits to enough sequences in the nr and IMG/VR databases for phylogenetic analysis (Supplemental File 1; Supplemental Table 6). This analysis identified vMAG001 (Adintoviridae), vMAG043 (Imitevirales), and vMAG060 (unknown taxonomy) as putatively host infecting, and vMAG055 (Megaviricetes) as putatively coral endosymbiont infecting.

### Assessing asymptomatic/resistant colonies

We assessed the relative abundance of our selected vMAGs and pMAGs in the putative asymptomatic HC (i.e. HC_1 and HC_2). Combined, these vMAGs made up 91.04% of the virome in HC_2 and 77.64% in colony HC_1. Similarly, we observe that pMAG01 had a relative abundance of 63.87% and 61.95% (respectively), and pMAG02 had a relative abundance of 2.52% and 5.76%.

### Validating MAG signatures using data from the Florida SCTLD outbreak

SCTLD metagenomic data from Rosalses *et al*. [28] was analyzed for the presence of our selected SCTLD-associated MAGs, across the four targeted species: *S. intersepta*, *D. labyrinthiformis*, *D. stokesii*, and *M. meandrites*. Fourteen vMAGs (generated in this study) were present in the samples from Florida, which included all five of our SCTLD-associated vMAGs (Fig. 3C). Two vMAGs were exclusive to *D. labyrinthiformis* (from Florida), two were detected in two coral species (including vMAG043), four were detected in three coral species (including vMAG001, vMAG058, and vMAG060), and six were detected in all four coral species (including vMAG055). Note, the vMAGs identified in the Florida samples that do form not part of our selected set had low relative abundances (~ 0.001) in the data generated from the Dominican Republic (this study). A log_2_TPM based trend analysis was done to assess variation based on health states but was deemed unreliable due to the unknown outcomes of the HC (Supplemental Fig. 5; Supplemental Table 7).

Of the generated 85 pMAGs in this study, only three were present in the Florida data. No pMAG was detected in more than two coral species. Of the SCTLD-associated pMAGs (generated in this study), pMAG01 was present only in *D. labyrinthiformis*, whereas pMAG02 was not detected in any species (Fig. 3C). pMAG84, annotated as *Bacterioplanoides* sp024397975 (class: Gammaproteobacteria), was detected only in *S. intersepta,* and pMAG50, annotated as *Parasynechococcus* sp002724845 (class: Cyanobacteria), was detected in *S. intersepta* and *M. meandrites*.

## Discussion

### Marked microbiome and endosymbiont shifts exist, independent of coral health state

Significant efforts have been made to identify a causative agent of SCTLD, yet fulfilling Koch’s postulates in corals remains a challenge. Of the 18 known coral diseases, only five have satisfied Koch’s postulates, highlighting the challenges facing disease studies in this group [17]. Our research shows that SCTLD-affected corals have substantial shifts in their prokaryotic microbiome and virome. We observe a clear dispersion of microbial beta-diversity with the clustering of diseased colonies (AH and DL), which has been documented in the literature [20, 78]. Alpha-diversity metrics (Shannon diversity, chao1 richness, and Simpson index) demonstrate that HC maintain rich and diverse microbial profiles, which are potentially tailored to their specific micro-environments [79–81]. Consistent with previous studies, we also observe that upon infection, these microbiomes become more uniform, with reduced diversity, evenness, and richness *via* the loss of prokaryotic and bacteriophage communities [18, 78]. We also observe that upon lesion formation (i.e. DL), there is a slight increase in alpha diversity, suggesting a new (potentially opportunistic or pathogenic) microbiome has colonized the diseased colonies.

The current results suggest that microbiome uniformity is observable, even in the absence of visible SCTLD lesions. This outcome is found in samples HC_1 and HC_2, which were initially tagged as HC because no visible symptoms of SCTLD were observed during field sampling. Follow-up observations nine months after initial sample collection showed that these colonies were still alive with no visible signs of SCTLD. In addition, these HC samples had a high load of SCTLD-associated vMAGs, but a different pMAG composition when compared to the infected colonies. These colonies may therefore provide a novel case of asymptomatic/resistance to SCTLD infections, which has been suggested in previous studies [11, 29]. The presence of viruses, in both asymptomatic and symptomatic samples has been noted in sea-stars [82], however additional life history studies need to be performed to validate these results in SCTLD-affected corals. The HC_1 and HC_2 colonies may have had an innate immunity/resistance to the infection, despite containing SCTLD-associated vMAGs. The role of genotype has been well characterized in conferring thermal stress induced bleaching resilience in corals [83–85], but this has not yet been translated to coral diseases. However, preliminary results have shown variations in genotype resilience and susceptibility in other coral diseases such as White Syndrome and White Pox Disease [86–89]. Regardless, this finding demonstrates the potential for treatment of SCTLD and the selection of resistant genotypes. These observations underscore the urgent need to develop diagnostic biomarkers for SCTLD across various coral species to facilitate informed reef management practices.

### Algal endosymbiont composition in SCTLD affected corals

Four endosymbiont species were detected in the collected samples: *B. minutum*, *Coenocyathus goreaui*, *D. trenchii* (CCMP2556 and SCF082_1), and *S. necroappetens* (Supplemental Fig. 1, Supplemental Table 3). The presence of these endosymbionts is consistent with previous population studies in the Caribbean [90]. Whereas, *D. trenchii* and *B. minutum* are highly prevalent in affected colonies (AH and DL), the opportunistic *S. necroappetens*, which has been previously isolated from Yellow Band Disease affected corals (active lesions) and bleached colonies in the Caribbean, is also present [91]. Given that *S. necroappetens* has been previously isolated from other diseased and bleached corals, it is unlikely to play a direct, causative role in SCTLD, rather, this alga may be an opportunist that associates with unhealthy coral tissue.

### DNA viruses appear to play a key role in infection

We observe two clear trends in the virome, the first is marked by loss of the bacteriophage Caudoviricetes vMAGs, potentially linked to the concurrent loss of numerous pMAGs that may serve as hosts, highlighting the complex interplay between microbiome, virome, and coral host [79, 92, 93]. The second trend we observe is the significant increase in five selected SCTLD-associated vMAGs in diseased colonies. Whereas these vMAGs make up a small component of the virome of HC samples (~ 15%), they constitute a large proportion of the AH and DL samples (~ 91% and ~ 76%, respectively). Furthermore, these vMAGs comprise the majority of the virome in the asymptomatic/resistant colonies and are present in the four SCTLD-affected corals in Florida, suggesting a connection between these vMAGs and SCTLD.

Phylogenetic analysis of the proteins predicted in each SCTLD-associated vMAG was used to infer a putative viral host. Three of the five SCTLD-associated vMAGs contained genes whose phylogenies included coral- or hydra-associated viral sequences, supporting the possibility of direct infection of the coral host. One vMAG also included endosymbiont sequences (e.g. *Symbiodinium* spp.) in the gene trees, suggesting a potential interaction with the algal symbiont community. However, the frequent presence of prokaryotic sequences in many phylogenies complicates host prediction, as does the uncertain association of the viral sequences from IMG/VR which are interspersed throughout each tree, making it difficult to determine whether the viruses primarily infect the coral, its symbionts, or members of the microbiome. The limited functional annotation of viral genes in these vMAGs limits our ability to infer infection mechanisms.

### Prokaryotic composition confers resilience or susceptibility

Compared to the HC, most pMAGs showed decreased abundance in diseased colonies. In HC samples, Alphaproteobacteria had the highest abundance followed by Gammaproteobacteria, both of which have been identified in previous SCTLD studies [20, 27]. We observed a decline in the majority of pMAGs in diseased colonies relative to healthy, suggesting microbiome destabilization. Two candidate pMAGs had significant increases in diseased colonies, one representing Leptospirae and one, Gammaproteobacteria. In HC samples, these bacteria had extremely low (0.013%) median abundance in the microbiome, however combined, in AH samples these two MAGs comprise up to ~48% of the microbiome (Leptospirae ~30% and Gammaproteobacteria MAG ~ 18%). However, these pMAGs showed a clear decline in abundance in DL samples: i.e. upon tissue loss, suggesting a shift in the microbiome at this stage of disease progression. Gene content analysis of these two pMAGs revealed that Leptospirae encoded more virulence factor genes, which is unsurprising given that pathogenic species from the genus *Leptospira* cause leptospirosis in humans and utilize long-chain fatty acids (LFAs) as their sole carbon and energy source [94]. The Leptospirales MAG identified in this study contained several genes associated with pathogenicity, notably, *tlyA* and *tlyC*, which are hemolysin genes implicated in erythrocyte lysis in model organisms. In addition, key *fli* genes encoding components of the flagellar apparatus, structures that facilitate motility and contribute to host infection, were also present in our Leptospirales MAG [94]. Based on these findings, we hypothesize that this pMAG may be pathogenic, acquiring LFAs from the coral in the AH tissue, but upon tissue loss (DL tissue), declines in abundance with its host.

Our results point to three distinct microbiome phases: the first, a rich and diverse community found in HC, the second, a reduction in microbiome diversity upon SCTLD infection (tissue still present), and third, colonization by opportunistic/pathogenic microbes (increasing diversity) as the disease progresses. We suggest that many of the microbes involved in this community shift are always associated with the coral, however upon infection, the decline of the health dominant microbiome creates an open niche that opportunistic/pathogenic species exploit. Interestingly, in the two asymptomatic/resistant colonies (HC_1 and HC_2), we observed only marginal levels of Leptospirae. Instead, these colonies were dominated by pMAG01 (Gammaproteobacteria) (Fig. 2A and Supplemental Fig. 6), which had fewer virulence genes (relative to pMAG02). The relatively higher abundance of Gammaproteobacteria, when compared to the Leptospirae in these colonies may thus prevent the progression to symptomatic disease. Moreover, we speculate that the dominant Gammaproteobacteria present in these colonies may function as a natural probiotic (i.e. beneficial microbiome), offering colonization resistance against Leptospirae and thereby mitigating disease progression. We further highlight that Leptospirae has not been previously associated to SCTLD, supporting the role of prokaryotes as opportunistic pathogens during disease progression [5, 16, 19, 21, 78]. Other prokaryotes associated with SCTLD (e.g. Rhodobacterales, Rhizobiales*,* and *Vibrio* spp.) were not detected in our study or were present in extremely low abundances. This suggests that SCTLD-associated opportunistic pathogens may reflect local conditions [5, 89].

### Pathogenic MAG presence in other SCTLD datasets

The presence of our selected MAGs in other SCTLD-affected coral species was assessed using data from Rosales *et al*. [28] (BioProject PRJNA576217). All our selected vMAGs were detected in this analysis (Fig. 3C), with several found across multiple species, consistent with their role in SCTLD. This analysis turned up our selected vMAGs in the HC samples (see Supplemental Table 7). It is unclear whether the fate of HC was studied by the authors: i.e. whether any of them later developed SCTLD, preventing further assessment of these colonies as putative asymptomatic/resistant. We only detected three pMAGs in any of the four coral species from Florida, of which two were specific to a single species, and one (Cyanobacteria) was present in two species. Overall, we see a more conserved virus distribution in SCTLD-affected coral species than of prokaryotes. This finding suggests that the prokaryote communities associated with this disease are highly geography and host species specific, aligning with the opportunistic pathogen role previously attributed to SCTLD associated prokaryotes [5, 28, 78, 89]. Nonetheless, the detection of SCTLD-associated vMAGs in multiple coral species, collected over 1000 miles away and several years apart in time, highlights their potential significance in coral disease dynamics.

### A working multi-kingdom model for SCTLD progression

**Figure 4:**
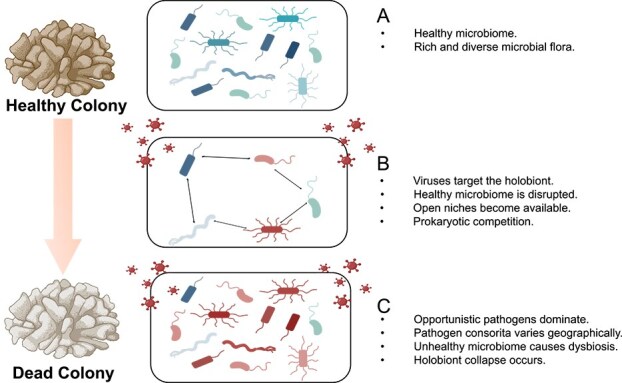
**Working model for SCTLD infection. (A)** A healthy coral holobiont with a rich and diverse microbiome. **(B)** Viruses target components of the healthy holobiont, which either directly or indirectly kill most prokaryotes, opening a niche. (**C**) Previously low abundance bacteria and other opportunistic pathogens flourish and dominate the coral holobiont post-viral infection. Microbiome dysbiosis, caused by the primary viral infection, results in a significant shift in the prokaryotic microbiome towards one dominated by pathogenic bacteria, which is the predominant cause of lesion formation and visual SCTLD symptoms.

Based on our findings and the results of previous studies, we propose a multi-kingdom model for the progression of SCTLD (Fig. 4), which may make it challenging to fulfill Koch’s postulates (i.e. which focuses on a specific agent) for this disease. The detection and significant enrichment of vMAGs in SCTLD-affected samples across different studies, geographic locations, and coral species suggest a pivotal role for viruses in disease progression. We hypothesize that these viruses infect microbial members of the coral holobiont, triggering dysbiosis and a collapse of the native microbiome, perhaps through the activation of the coral host immune response [5, 89, 95, 96]. This destabilization creates an open ecological niche which opportunistic microbes can occupy. Leptospirae and Gammaproteobacteria apparently filled this role in our dataset, clearly dominating the microbiomes of affected and asymptomatic colonies. These opportunistic microbes may harbor virulence factors (e.g. hemolysins) which cause tissue degradation and contribute to coral mortality. We note that the Leptospirae was not detected in the Florida study, and the Gammaproteobacteria was limited to *D. labyrinthiformis.* Consistent with previous work, we suggest that opportunistic pathogens may comprise polymicrobial consortia that reflect geographic location and coral host [5, 11, 18, 20, 28, 78].

Lastly, we highlight the presence of potentially SCTLD asymptomatic/resistant coral colonies, which has been noted in other coral diseases [89, 95]. Apart from putative host resistance, we suggest a microbial resilience hypothesis. Upon microbiome destabilization, if a non-pathogenic taxon (e.g. Gammaproteobacteria in this study) comes to dominate, then it may act beneficially, inhibiting colonization of pathogenic species and promoting disease resilience [93, 97]. This working model aligns with the observed success of probiotic and antibiotic treatments in halting lesion progression in infected colonies [22–25], despite the lack of clear causative prokaryotic agents. That is, anti-microbial interventions may mitigate secondary bacterial infections, allowing the coral host to recover, or the microbiome to stabilize despite an ongoing viral presence.

### Study limitations and future directions

We recognize several important limitations in this study. First, because the viruses of interest have not yet been isolated, it is impossible to assess host specificity. In addition, the small genome size of many vMAGs suggests they may be incomplete, limiting functional interpretation. Nevertheless, the consistent increase in abundance of these vMAGs in diseased colonies and their presence in other SCTLD datasets suggests they likely play an important role in disease dynamics. Additional research is needed to better resolve the host range and infection mechanisms of these viruses, ideally through targeted isolation and improved genome completeness. Despite these limitations, our initial findings support the idea that DNA viruses are likely to be significant contributors to SCTLD infection.

Another important outcome of our work is the identification of putative asymptomatic/ resistant colonies, which is a novel finding for SCTLD. However, this inference requires validation through long-term life history monitoring, which was beyond the scope of this study and challenging, given the ubiquity of the disease throughout the Caribbean. Future SCTLD and coral disease studies should account for the potential presence of such asymptomatic/resistant states and investigate outcomes for both diseased and HC. Host coral-targeted transcriptomic or proteomic studies may provide key insights into the basis of coral resilience. We emphasize that this study does not fulfill Koch’s postulates (which may prove challenging for SCTLD), but rather we propose a working model of a multi-kingdom infection involving both viral and prokaryote components. If correct, it may prove challenging to develop in situ treatment of SCTLD given the complex etiology. Additional experimental work is needed to establish causality, identify putative viral targets, and generate complete genome data for characterization. Nonetheless, the vMAGs identified here serve as valuable early biomarkers for predicting infection risk or resistance in coral colonies.

## Supplementary Material

Supplemental_Figure_1_ycaf226

Supplemental_Figure_2_ycaf226

Supplemental_figure_3_ycaf226

Supplemental_Figure_4_ycaf226

Supplemental_Figure_5_R1_ycaf226

Supplemental_Figure_6_ycaf226

Supplemental_Table_1_ycaf226

Supplemental_Table_2_ycaf226

Supplemental_Table_3_ycaf226

Supplemental_Table_4_ycaf226

Supplemental_Table_5_ycaf226

Supplemental_Table_6_ycaf226

Supplemental_Table_7_ycaf226

Supplemental_Table_8_ycaf226

ISMECOMMUN_Supplemental_Text_R1_ycaf226

## Data Availability

Raw data is available on NCBI under the project number PRJNA1278695. All code used in this study is available on https://github.com/shrinivas-nandi/shrinivas-nandi/tree/main/Projects/Dominican_Republic_SCLTD. The Naïve ATLAS pipeline and code is available on https://github.com/TimothyStephens/naive_atlas.

## References

[1] Green EP, Bruckner AW. The significance of coral disease epizootiology for coral reef conservation. *Biol Conserv* 2000;96:347–61. 10.1016/S0006-3207(00)00073-2

[2] Randazzo-Eisemann Á, Garza-Pérez JR, Figueroa-Zavala B. The role of coral diseases in the flattening of a Caribbean coral reef over 23 years. *Mar Pollut Bull* 2022;181:113855. 10.1016/j.marpolbul.2022.113855 35753248

[3] Precht WF, Gintert BE, Robbart ML. et al. Unprecedented disease-related coral mortality in Southeastern Florida. *Sci Rep* 2016;6:31374. 10.1038/srep31374 27506875 PMC4979204

[4] Studivan MS, Rossin AM, Rubin E. et al. Reef sediments can act as a stony coral tissue loss disease vector. *Front Mar Sci* 2022;8:815698. 10.3389/fmars.2021.815698

[5] Papke E, Carreiro A, Dennison C. et al. Stony coral tissue loss disease: a review of emergence, impacts, etiology, diagnostics, and intervention. *Front Mar Sci* 2024;10:1321271. 10.3389/fmars.2023.1321271

[6] Camacho-Vite C, Estrada-Saldívar N, Pérez-Cervantes E. et al. Differences in the progression rate of SCTLD in *Pseudodiploria strigosa* are related to colony size and morphology. *Front Mar Sci* 2022;9:790818. 10.3389/fmars.2022.790818

[7] Muller EM, Sartor C, Alcaraz NI. et al. Spatial epidemiology of the stony-coral-tissue-loss disease in Florida. *Front Mar Sci* 2020;7:163. 10.3389/fmars.2020.00163

[8] Dobbelaere T, Muller EM, Gramer LJ. et al. Coupled Epidemio-hydrodynamic Modeling to understand the spread of a deadly coral disease in Florida. *Front Mar Sci* 2020;7:591881. 10.3389/fmars.2020.591881

[9] Huntley N, Brandt ME, Becker CC. et al. Experimental transmission of stony coral tissue loss disease results in differential microbial responses within coral mucus and tissue. *ISME Communications* 2022;2:46. 10.1038/s43705-022-00126-3 37938315 PMC9723713

[10] Rosenau NA, Gignoux-Wolfsohn S, Everett RA. et al. Considering commercial vessels as potential vectors of stony coral tissue loss disease. *Front Mar Sci* 2021;8:709764. 10.3389/fmars.2021.709764 PMC9175181 35685121

[11] Evans JS, Paul VJ, Kellogg CA. Biofilms as potential reservoirs of stony coral tissue loss disease. *Front Mar Sci* 2022;9:1009407. 10.3389/fmars.2022.1009407

[12] Brandt ME, Ennis RS, Meiling SS. et al. The emergence and initial impact of stony coral tissue loss disease (SCTLD) in the United States Virgin Islands. *Front Mar Sci* 2021;8:715329. 10.3389/fmars.2021.715329

[13] Cróquer A, Zambrano S, King Pérez SA. et al. Stony coral tissue loss disease and other diseases affect adults and recruits of major reef builders at different spatial scales in the Dominican Republic. *GCR* 2022;33:GCFI1–13. 10.18785/gcr.3301.03

[14] Alvarez-Filip L, Estrada-Saldívar N, Pérez-Cervantes E. et al. A rapid spread of the stony coral tissue loss disease outbreak in the Mexican Caribbean. *PeerJ* 2019;7:e8069. 10.7717/peerj.8069 31788355 PMC6883952

[15] Villalpando MF, Guendulain-García SD, Valdez-Trinidad A. et al. Coral reefs of southeastern Dominican Republic hit by two simultaneous epizootic events. *bms* 2022;98:507–8. 10.5343/bms.2022.0015

[16] Stony Coral Tissue Loss Disease (SCTLD) . Coral Disease & Health Consortium, https://cdhc.noaa.gov/coral-disease/characterized-diseases/stony-coral-tissue-loss-disease-sctld/.

[17] Sutherland K, Porter J, Torres C. Disease and immunity in Caribbean and Indo-Pacific zooxanthellate corals. *Mar Ecol Prog Ser* 2004;266:273–302. 10.3354/meps266273

[18] Evans JS, Paul VJ, Ushijima B. et al. Investigating microbial size classes associated with the transmission of stony coral tissue loss disease (SCTLD). *PeerJ* 2023;11:e15836. 10.7717/peerj.15836 37637172 PMC10460154

[19] Clark AS, Williams SD, Maxwell K. et al. Characterization of the microbiome of corals with stony coral tissue loss disease along Florida’s coral reef. *Microorganisms* 2021;9:2181. 10.3390/microorganisms9112181 34835306 PMC8623284

[20] Arriaga-Piñón ZP, Aguayo-Leyva JE, Álvarez-Filip L. et al. Microbiomes of three coral species in the Mexican Caribbean and their shifts associated with the stony coral tissue loss disease. *PLoS One* 2024;19:e0304925. 10.1371/journal.pone.0304925 39186575 PMC11346732

[21] Rosales SM, Clark AS, Huebner LK. et al. Rhodobacterales and Rhizobiales are associated with stony coral tissue loss disease and its suspected sources of transmission. *Front Microbiol* 2020;11:681. 10.3389/fmicb.2020.00681 32425901 PMC7212369

[22] Neely KL, Macaulay KA, Hower EK. et al. Effectiveness of topical antibiotics in treating corals affected by stony coral tissue loss disease. *PeerJ* 2020;8:e9289. 10.7717/peerj.9289 32551200 PMC7292019

[23] Forrester GE, Arton L, Horton A. et al. Antibiotic treatment ameliorates the impact of stony coral tissue loss disease (SCTLD) on coral communities. *Front Mar Sci* 2022;9:9. 10.3389/fmars.2022.859740

[24] Forrester GE, Arton L, Horton A. et al. The relative effectiveness of chlorine and antibiotic treatments for stony coral tissue loss disease. *Front Mar Sci* 2024;11:1465173. 10.3389/fmars.2024.1465173

[25] Ushijima B, Gunasekera SP, Meyer JL. et al. Chemical and genomic characterization of a potential probiotic treatment for stony coral tissue loss disease. *Commun Biol* 2023;6:248. 10.1038/s42003-023-04590-y 37024599 PMC10079959

[26] Demko AM, Sneed JM, Houk LJ. et al. The effects of probiotics and stony coral tissue loss disease exposure on coral recruits. *Coral Reefs* 2025;44:411–22. 10.1007/s00338-024-02610-9

[27] Meyer JL, Castellanos-Gell J, Aeby GS. et al. Microbial community shifts associated with the ongoing stony coral tissue loss disease outbreak on the Florida reef tract. *Front Microbiol* 2019;10:2244. 10.3389/fmicb.2019.02244 31608047 PMC6769089

[28] Rosales SM, Huebner LK, Clark AS. et al. Bacterial metabolic potential and micro-eukaryotes enriched in stony coral tissue loss disease lesions. *Front Mar Sci* 2022;8:776859. 10.3389/fmars.2021.776859

[29] Work TM, Weatherby TM, Landsberg JH. et al. Viral-like particles are associated with endosymbiont pathology in Florida corals affected by stony coral tissue loss disease. *Front Mar Sci* 2021;8:750658. 10.3389/fmars.2021.750658

[30] Beavers KM, Van Buren EW, Rossin AM. et al. Stony coral tissue loss disease induces transcriptional signatures of in situ degradation of dysfunctional Symbiodiniaceae. *Nat Commun* 2023;14:2915. 10.1038/s41467-023-38612-4 37217477 PMC10202950

[31] Veglia AJ, Beavers K, Van Buren EW. et al. Alphaflexivirus genomes in stony coral tissue loss disease-affected, disease-exposed, and disease-unexposed coral colonies in the U.S. *Virgin Islands Microbiol Resour Announc* 2022;11:e01199–21. 10.1128/mra.01199-21 35175123 PMC8852308

[32] Heinz JM, Lu J, Huebner LK. et al. Novel metagenomics analysis of stony coral tissue loss disease. *G3* 2024;14:jkae137. 10.1093/g3journal/jkae137 38900914 PMC11304949

[33] Howe-Kerr LI, Knochel AM, Meyer MD. et al. Filamentous virus-like particles are present in coral dinoflagellates across genera and ocean basins. *The ISME Journal* 2023;17:2389–402. 10.1038/s41396-023-01526-6 37907732 PMC10689786

[34] Work TM, Miller J, Kelley T. et al. Pathology of lesions in corals from the US Virgin Islands after emergence of stony coral tissue loss disease. *Coral Reefs* 2025;44:179–92. 10.1007/s00338-024-02595-5

[35] Aeby GS, Ushijima B, Campbell JE. et al. Pathogenesis of a tissue loss disease affecting multiple species of corals along the Florida reef tract. *Front Mar Sci* 2019;6:678. 10.3389/fmars.2019.00678

[36] Kieser S, Brown J, Zdobnov EM. et al. ATLAS: a Snakemake workflow for assembly, annotation, and genomic binning of metagenome sequence data. *BMC Bioinformatics* 2020;21:257. 10.1186/s12859-020-03585-4 32571209 PMC7310028

[37] Espinoza JL, Phillips A, Prentice MB. et al. Unveiling the microbial realm with VEBA 2.0: a modular bioinformatics suite for end-to-end genome-resolved prokaryotic, (micro)eukaryotic and viral multi-omics from either short- or long-read sequencing. *Nucleic Acids Res* 2024;52:e63–3. 10.1093/nar/gkae528 38909293 PMC11317156

[38] Li D, Liu CM, Luo R. et al. MEGAHIT: an ultra-fast single-node solution for large and complex metagenomics assembly via succinct de Bruijn graph. *Bioinformatics* 2015;31:1674–6. 10.1093/bioinformatics/btv033 25609793

[39] Li H . New strategies to improve minimap2 alignment accuracy. *Bioinformatics* 2021;37:4572–4. 10.1093/bioinformatics/btab705 34623391 PMC8652018

[40] Kang DD, Li F, Kirton E. et al. MetaBAT 2: an adaptive binning algorithm for robust and efficient genome reconstruction from metagenome assemblies. *PeerJ* 2019;7:e7359. 10.7717/peerj.7359 31388474 PMC6662567

[41] Wu Y-W, Simmons BA, Singer SW. MaxBin 2.0: an automated binning algorithm to recover genomes from multiple metagenomic datasets. *Bioinformatics* 2016;32:605–7. 10.1093/bioinformatics/btv638 26515820

[42] Sieber CMK, Probst AJ, Sharrar A. et al. Recovery of genomes from metagenomes via a dereplication, aggregation and scoring strategy. *Nat Microbiol* 2018;3:836–43. 10.1038/s41564-018-0171-1 29807988 PMC6786971

[43] Pronk LJU, Medema MH. Whokaryote: distinguishing eukaryotic and prokaryotic contigs in metagenomes based on gene structure. *Microbial Genomics* 2022;8:8. 10.1099/mgen.0.000823 PMC9465069 35503723

[44] Vollmers J, Wiegand S, Lenk F. et al. How clear is our current view on microbial dark matter? (Re-)assessing public MAG & SAG datasets with MDMcleaner. *Nucleic Acids Res* 2022;50:e76. 10.1093/nar/gkac294 35536293 PMC9303271

[45] Chklovski A, Parks DH, Woodcroft BJ. et al. CheckM2: a rapid, scalable and accurate tool for assessing microbial genome quality using machine learning. *Nat Methods* 2023;20:1203–12. 10.1038/s41592-023-01940-w 37500759

[46] Chklovski A, Parks DH, Woodcroft BJ. et al. Author correction: CheckM2: a rapid, scalable and accurate tool for assessing microbial genome quality using machine learning. *Nat Methods* 2024;21:735–5. 10.1038/s41592-024-02248-z 38514780

[47] Shaw J, Yu YW. Fast and robust metagenomic sequence comparison through sparse chaining with skani. *Nat Methods* 2023;20:1661–5. 10.1038/s41592-023-02018-3 37735570 PMC10630134

[48] Chaumeil PA, Mussig AJ, Hugenholtz P. et al. GTDB-Tk: a toolkit to classify genomes with the genome taxonomy database. *Bioinformatics* 2020;36:1925–7. 10.1093/bioinformatics/btz848 PMC7703759 31730192

[49] Manni M, Berkeley MR, Seppey M. et al. BUSCO update: novel and streamlined workflows along with broader and deeper phylogenetic coverage for scoring of eukaryotic, prokaryotic, and viral genomes. *Mol Biol Evol* 2021;38:4647–54. 10.1093/molbev/msab199 34320186 PMC8476166

[50] Camargo AP, Roux S, Schulz F. et al. Identification of mobile genetic elements with geNomad. *Nat Biotechnol* 2023;42:1303–12. 10.1038/s41587-023-01953-y 37735266 PMC11324519

[51] Schwengers O, Jelonek L, Dieckmann MA. et al. Bakta: rapid and standardized annotation of bacterial genomes via alignment-free sequence identification. *Microbial Genomics* 2021;7:000685. 10.1099/mgen.0.000685 34739369 PMC8743544

[52] Seemann T . Prokka: rapid prokaryotic genome annotation. *Bioinformatics* 2014;30:2068–9. 10.1093/bioinformatics/btu153 24642063

[53] Laetsch DR, Blaxter ML. BlobTools: interrogation of genome assemblies. *F1000Res* 2017;6:1287. 10.12688/f1000research.12232.1

[54] Shoguchi E, Beedessee G, Tada I. et al. Two divergent *Symbiodinium* genomes reveal conservation of a gene cluster for sunscreen biosynthesis and recently lost genes. *BMC Genomics* 2018;19:458. 10.1186/s12864-018-4857-9 29898658 PMC6001144

[55] Aranda M, Li Y, Liew YJ. et al. Genomes of coral dinoflagellate symbionts highlight evolutionary adaptations conducive to a symbiotic lifestyle. *Sci Rep* 2016;6:39734. 10.1038/srep39734 28004835 PMC5177918

[56] Shoguchi E, Shinzato C, Kawashima T. et al. Draft assembly of the *Symbiodinium minutum* nuclear genome reveals dinoflagellate gene structure. *Curr Biol* 2013;23:1399–408. 10.1016/j.cub.2013.05.062 23850284

[57] Liu H, Stephens TG, González-Pech RA. et al. *Symbiodinium* genomes reveal adaptive evolution of functions related to coral-dinoflagellate symbiosis. *Commun Biol* 2018;1:95. 10.1038/s42003-018-0098-3 30271976 PMC6123633

[58] González-Pech RA, Stephens TG, Chen Y. et al. Comparison of 15 dinoflagellate genomes reveals extensive sequence and structural divergence in family Symbiodiniaceae and genus *Symbiodinium*. *BMC Biol* 2021;19:73. 10.1186/s12915-021-00994-6 33849527 PMC8045281

[59] Dougan KE, Bellantuono AJ, Kahlke T. et al. Whole-genome duplication in an algal symbiont bolsters coral heat tolerance. *Sci Adv* 2024;10:eadn2218. 10.1126/sciadv.adn2218 39028812 PMC11259175

[60] Shah S, Dougan KE, Chen Y. et al. Massive genome reduction predates the divergence of Symbiodiniaceae dinoflagellates. *The ISME Journal* 2024;18:wrae059. 10.1093/ismejo/wrae059 38655774 PMC11114475

[61] Stephens TG, González-Pech RA, Cheng Y. et al. Genomes of the dinoflagellate *Polarella glacialis* encode tandemly repeated single-exon genes with adaptive functions. *BMC Biol* 2020;18:56. 10.1186/s12915-020-00782-8 32448240 PMC7245778

[62] Langmead B, Salzberg SL. Fast gapped-read alignment with bowtie 2. *Nat Methods* 2012;9:357–9. 10.1038/nmeth.1923 22388286 PMC3322381

[63] A Aroney STN, Newell RJP, Nissen JN. et al. CoverM: read alignment statistics for metagenomics. *Bioinformatics* 2025;41:btaf147. 10.1093/bioinformatics/btaf147 40193404 PMC11993303

[64] Gloor GB, Macklaim JM, Pawlowsky-Glahn V. et al. Microbiome datasets are compositional: and this is not optional. *Front Microbiol* 2017;8:2224. 10.3389/fmicb.2017.02224 29187837 PMC5695134

[65] Calle ML . Statistical analysis of metagenomics data. *Genomics Inform* 2019;17:e6. 10.5808/GI.2019.17.1.e6 30929407 PMC6459172

[66] Xia Y . Statistical normalization methods in microbiome data with application to microbiome cancer research. *Gut Microbes* 2023;15:2244139. 10.1080/19490976.2023.2244139 37622724 PMC10461514

[67] Lin H, Peddada SD. Multigroup analysis of compositions of microbiomes with covariate adjustments and repeated measures. *Nat Methods* 2024;21:83–91. 10.1038/s41592-023-02092-7 38158428 PMC10776411

[68] Fernandes AD, Reid JN, Macklaim JM. et al. Unifying the analysis of high-throughput sequencing datasets: characterizing RNA-seq, 16S rRNA gene sequencing and selective growth experiments by compositional data analysis. *Microbiome* 2014;2:15. 10.1186/2049-2618-2-15 24910773 PMC4030730

[69] Robinson MD, McCarthy DJ, Smyth GK. edgeR : a Bioconductor package for differential expression analysis of digital gene expression data. *Bioinformatics* 2010;26:139–40. 10.1093/bioinformatics/btp616 19910308 PMC2796818

[70] Nearing JT, Douglas GM, Hayes MG. et al. Microbiome differential abundance methods produce different results across 38 datasets. *Nat Commun* 2022;13:342. 10.1038/s41467-022-28034-z 35039521 PMC8763921

[71] Weiss S, Xu ZZ, Peddada S. et al. Normalization and microbial differential abundance strategies depend upon data characteristics. *Microbiome* 2017;5:27. 10.1186/s40168-017-0237-y 28253908 PMC5335496

[72] Muzellec B, Teleńczuk M, Cabeli V. et al. PyDESeq2: a python package for bulk RNA-seq differential expression analysis. *Bioinformatics* 2023;39:btad547. 10.1093/bioinformatics/btad547 37669147 PMC10502239

[73] Buchfink B, Xie C, Huson DH. Fast and sensitive protein alignment using DIAMOND. *Nat Methods* 2015;12:59–60. 10.1038/nmeth.3176 25402007

[74] Katoh K, Misawa K, Kuma K. et al. MAFFT: a novel method for rapid multiple sequence alignment based on fast Fourier transform. *Nucleic Acids Res* 2002;30:3059–66. 10.1093/nar/gkf436 12136088 PMC135756

[75] Capella-Gutiérrez S, Silla-Martínez JM, Gabaldón T. trimAl: a tool for automated alignment trimming in large-scale phylogenetic analyses. *Bioinformatics* 2009;25:1972–3. 10.1093/bioinformatics/btp348 19505945 PMC2712344

[76] Kanehisa M, Sato Y, Morishima K. BlastKOALA and GhostKOALA: KEGG tools for functional characterization of genome and metagenome sequences. *J Mol Biol* 2016;428:726–31. 10.1016/j.jmb.2015.11.006 26585406

[77] Liu B, Zheng D, Zhou S. et al. VFDB 2022: a general classification scheme for bacterial virulence factors. *Nucleic Acids Res* 2022;50:D912–7. 10.1093/nar/gkab1107 34850947 PMC8728188

[78] Rosales SM, Huebner LK, Evans JS. et al. A meta-analysis of the stony coral tissue loss disease microbiome finds key bacteria in unaffected and lesion tissue in diseased colonies. *ISME Communications* 2023;3:19. 10.1038/s43705-023-00220-0 36894742 PMC9998881

[79] Mohamed AR, Ochsenkühn MA, Kazlak AM. et al. The coral microbiome: towards an understanding of the molecular mechanisms of coral–microbiota interactions. *FEMS Microbiol Rev* 2023;47:fuad005. 10.1093/femsre/fuad005 36882224 PMC10045912

[80] Bourne DG, Morrow KM, Webster NS. Insights into the coral microbiome: underpinning the health and resilience of reef ecosystems. *Ann Rev Microbiol* 2016;70:317–40. 10.1146/annurev-micro-102215-095440 27482741

[81] van Oppen MJH, Blackall LL. Coral microbiome dynamics, functions and design in a changing world. *Nat Rev Microbiol* 2019;17:557–67. 10.1038/s41579-019-0223-4 31263246

[82] Jackson EW, Wilhelm RC, Johnson MR. et al. Diversity of sea star-associated Densoviruses and transcribed endogenous viral elements of Densovirus origin. *J Virol* 2020;95:e01594–20. 10.1128/JVI.01594-20 32967964 PMC7737747

[83] Edmunds PJ . Evidence that reef-wide patterns of coral bleaching may be the result of the distribution of bleaching-susceptible clones. *Mar Biol* 1994;121:137–42. 10.1007/BF00349482

[84] Chille EE, Stephens TG, Misri D. et al. Gene expression response under thermal stress in two Hawaiian corals is dominated by ploidy and genotype. *Ecology and Evolution* 2024;14:e70037. 10.1002/ece3.70037 39050655 PMC11268936

[85] Fitt WK, Gates RD, Hoegh-Guldberg O. et al. Response of two species of Indo-Pacific corals, *Porites cylindrica* and *Stylophora pistillata*, to short-term thermal stress: the host does matter in determining the tolerance of corals to bleaching. *J Exp Mar Biol Ecol* 2009;373:102–10. 10.1016/j.jembe.2009.03.011

[86] Muller EM, van Woesik R, van Woesik R. Genetic susceptibility, Colony size, and water temperature drive white-pox disease on the coral *Acropora palmata*. *PLoS One* 2014;9:e110759. 10.1371/journal.pone.0110759 25372835 PMC4220941

[87] Muller EM, Bartels E, Baums IB. Bleaching causes loss of disease resistance within the threatened coral species *Acropora cervicornis*. *eLife* 7:e35066. 10.7554/eLife.35066 30203745 PMC6133546

[88] Lozada-Misa P, Kerr A, Raymundo L. Contrasting lesion dynamics of white syndrome among the scleractinian corals Porites spp. *PLoS One* 2015;10:e0129841. 10.1371/journal.pone.0129841 26120844 PMC4488276

[89] Vega Thurber R, Mydlarz LD, Brandt M. et al. Deciphering coral disease dynamics: integrating host, microbiome, and the changing environment. *Front Ecol Evol* 2020;8:575927. 10.3389/fevo.2020.575927

[90] Kennedy EV, Tonk L, Foster NL. et al. *Symbiodinium* biogeography tracks environmental patterns rather than host genetics in a key Caribbean reef-builder. *Orbicella annularis Proc Biol Sci* 2016;283:20161938. 10.1098/rspb.2016.1938 27807263 PMC5124097

[91] LaJeunesse TC, Lee SY, Gil-Agudelo DL. et al. *Symbiodinium necroappetens sp. nov*. (Dinophyceae): an opportunist ‘zooxanthella’ found in bleached and diseased tissues of Caribbean reef corals. *Eur J Phycol* 2015;50:223–38. 10.1080/09670262.2015.1025857

[92] Ambalavanan L, Iehata S, Fletcher R. et al. A review of marine viruses in coral ecosystem. *J Mar Sci Eng* 2021;9:711. 10.3390/jmse9070711

[93] Voolstra CR, Raina JB, Dörr M. et al. The coral microbiome in sickness, in health and in a changing world. *Nat Rev Microbiol* 2024;22:460–75. 10.1038/s41579-024-01015-3 38438489

[94] Evangelista KV, Coburn J. *Leptospira* as an emerging pathogen: A review of its biology, pathogenesis and host immune responses. *Future Microbiol* 2010;5:1413–25. 10.2217/fmb.10.102 20860485 PMC3037011

[95] Libro S, Vollmer SV. Genetic signature of resistance to white band disease in the Caribbean staghorn coral *Acropora cervicornis*. *PLoS One* 2016;11:e0146636. 10.1371/journal.pone.0146636 26784329 PMC4718514

[96] Libro S, Kaluziak ST, Vollmer SV. RNA-seq profiles of immune related genes in the staghorn coral *Acropora cervicornis* infected with white band disease. *PLoS One* 2013;8:e81821. 10.1371/journal.pone.0081821 24278460 PMC3836749

[97] Peixoto RS, Rosado PM, Leite DCA. et al. Beneficial microorganisms for corals (BMC): proposed mechanisms for coral health and resilience. *Front Microbiol* 2017;8:341. 10.3389/fmicb.2017.00341 28326066 PMC5339234

