# Supplementary figures and images for: Shifts in the microbiome and virome are associated with stony coral tissue loss disease (SCTLD)
Source: ISME Commun. 2025 Nov 27;5(1):ycaf226. doi: 10.1093/ismeco/ycaf226 (PMC12743298; doi:10.1093/ismeco/ycaf226)

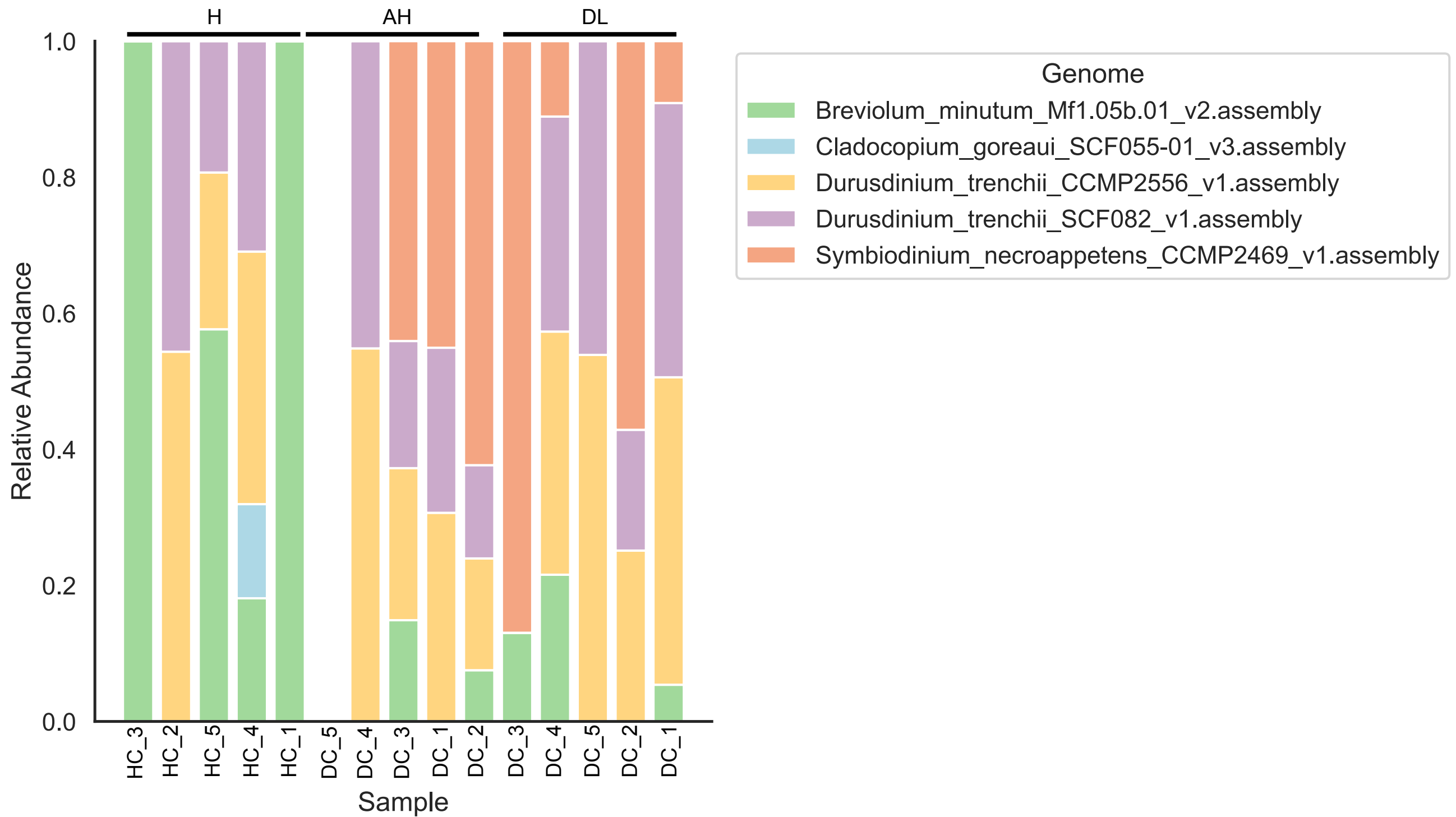

Supplement: Supplemental_Figure_1_ycaf226 [file supplemental_figure_1_ycaf226.pdf]

HC\_1

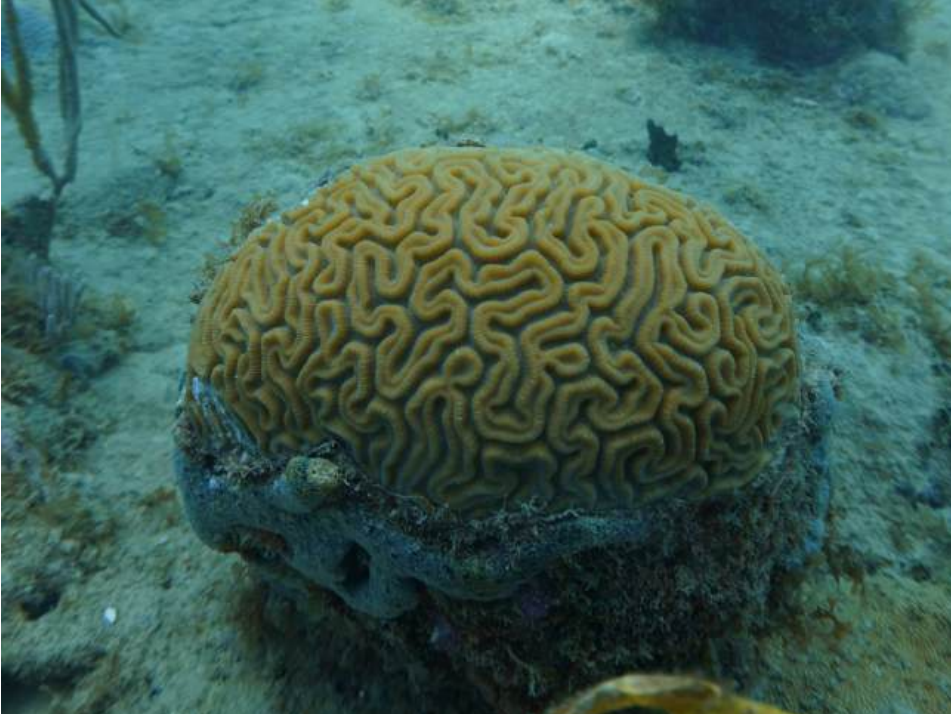

June 12, 2024

HC\_2

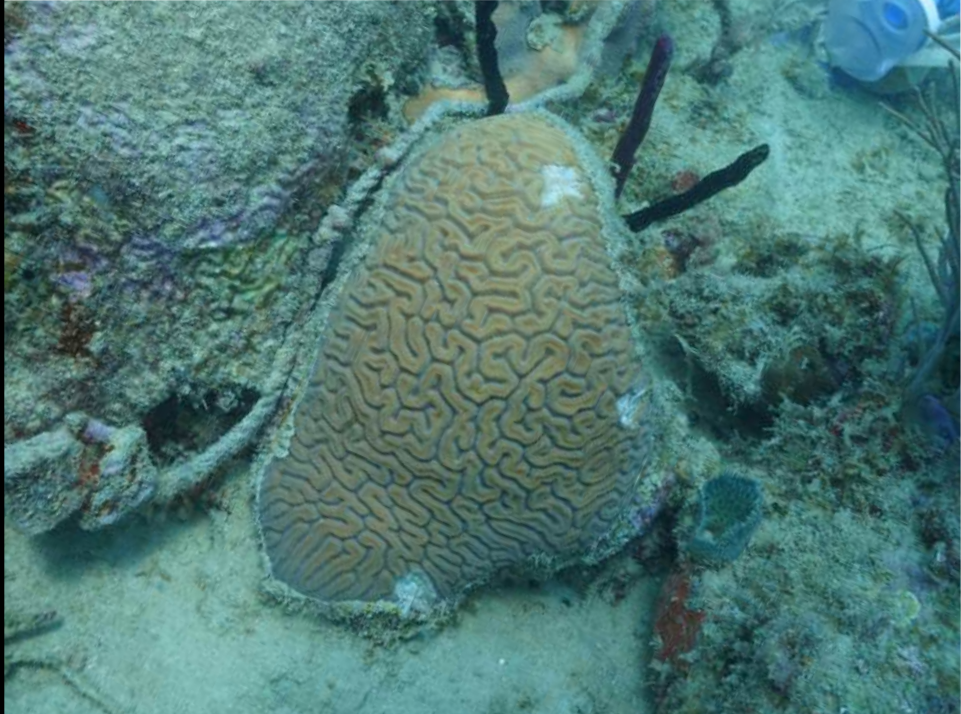

March 28, 2025

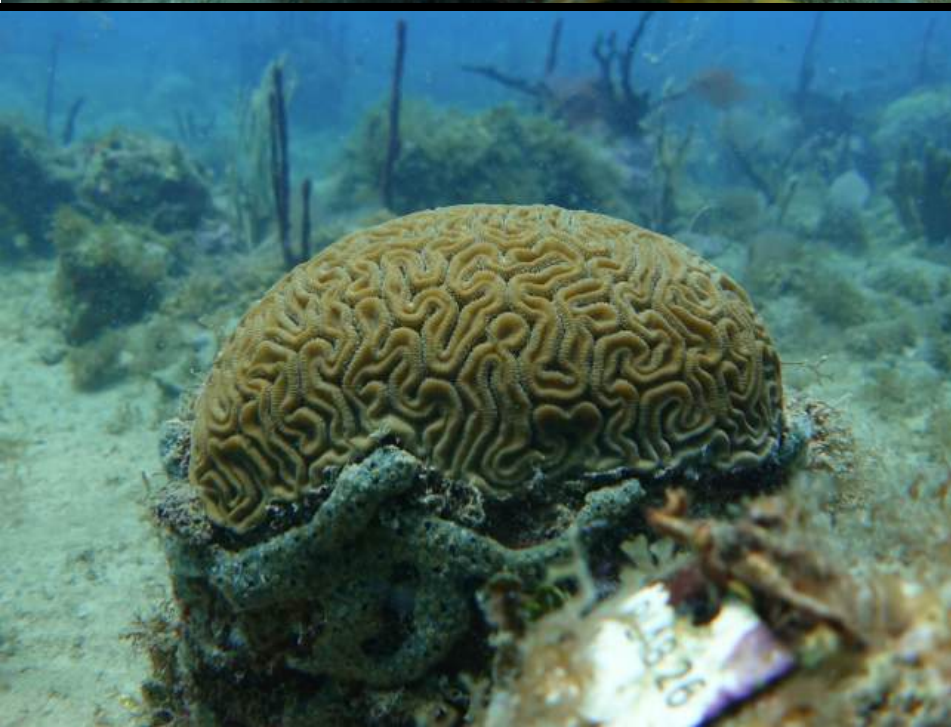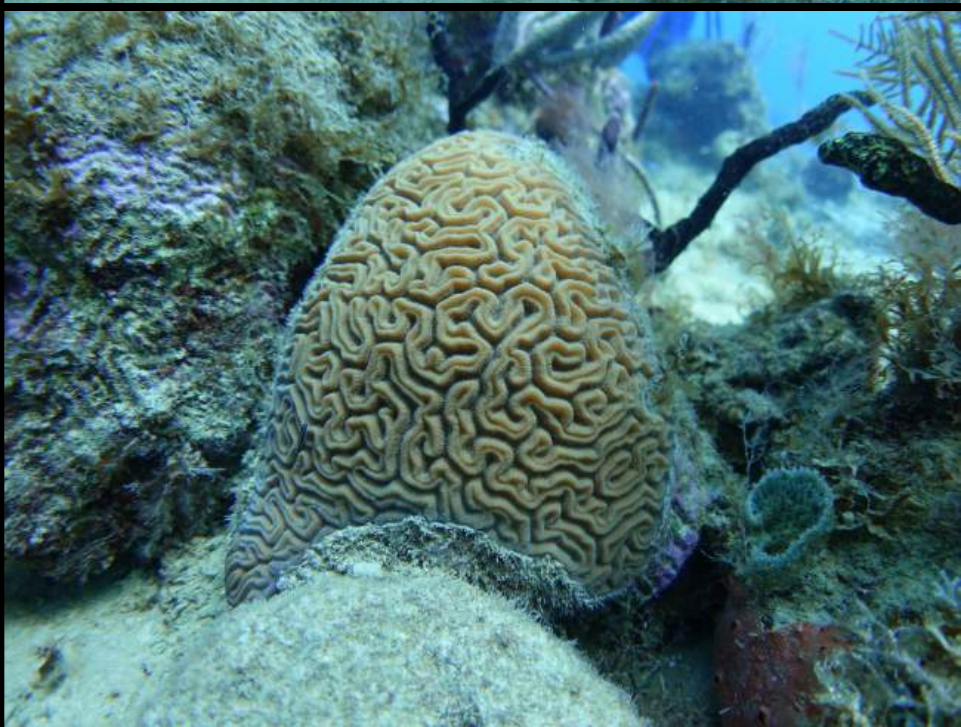

Supplement: Supplemental_Figure_2_ycaf226 [file supplemental_figure_2_ycaf226.pdf]

A

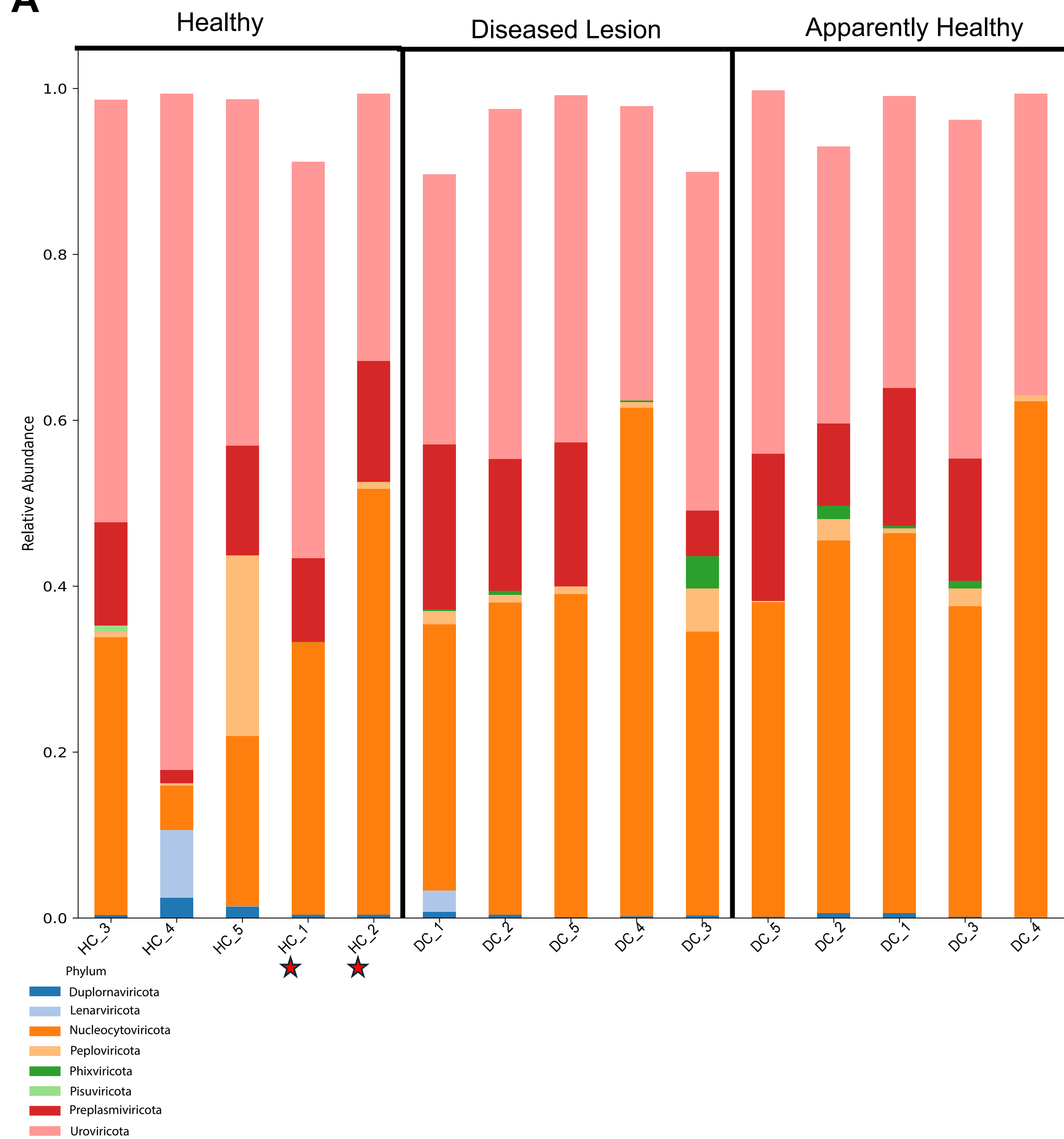

Supplement: Supplemental_figure_3_ycaf226 [file supplemental_figure_3_ycaf226.pdf]

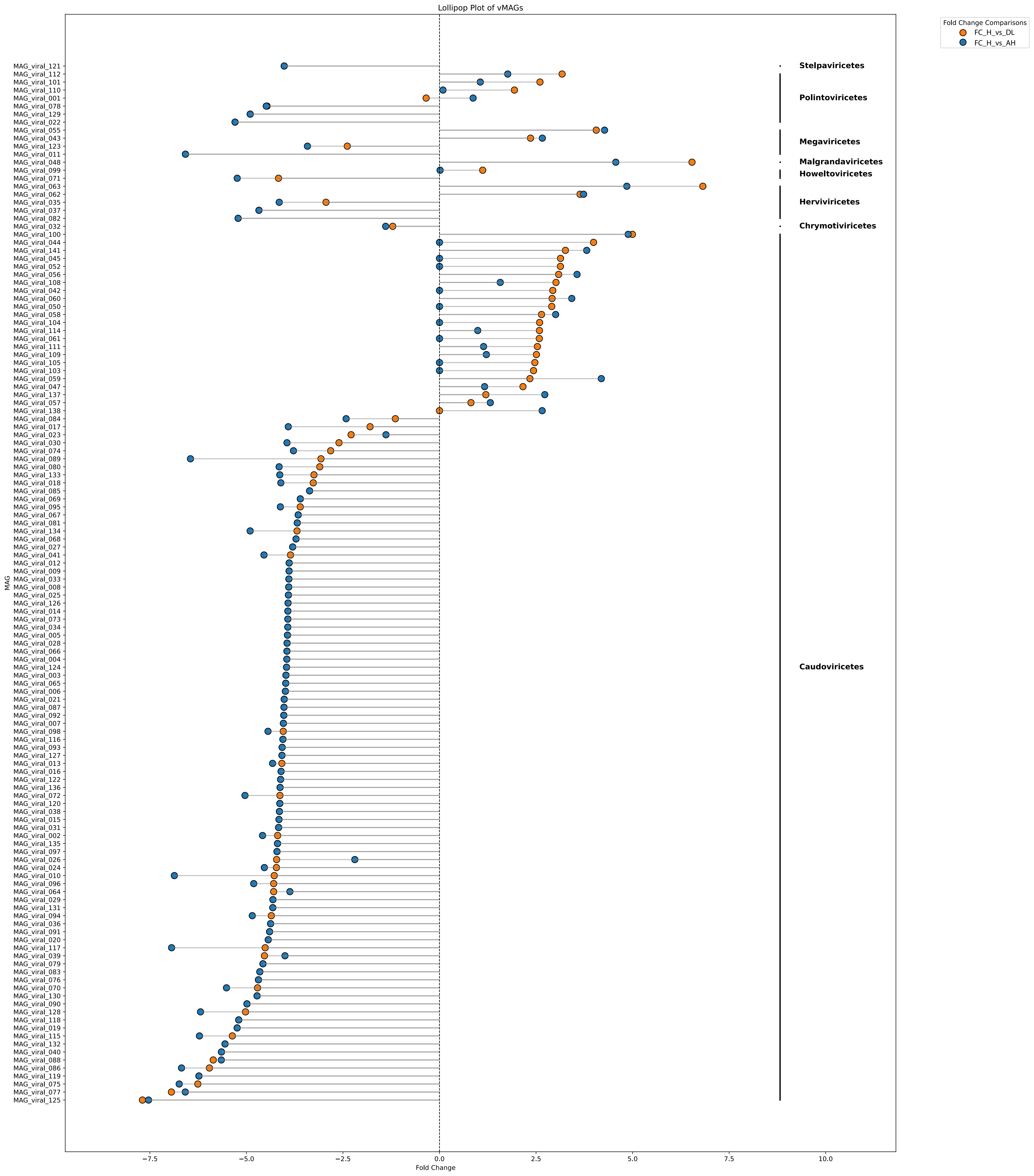

Supplement: Supplemental_Figure_4_ycaf226 [file supplemental_figure_4_ycaf226.pdf]

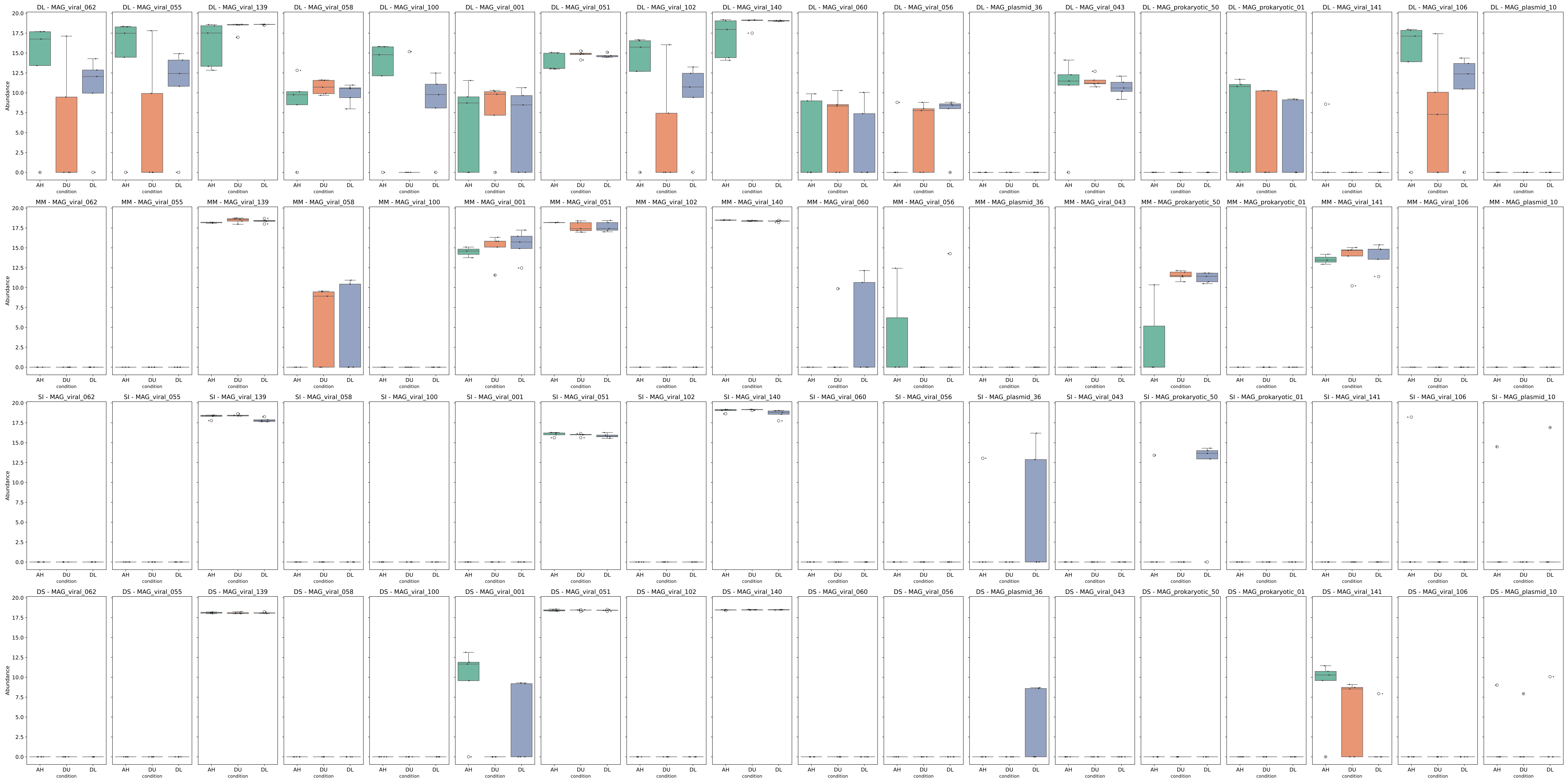

Supplement: Supplemental_Figure_5_R1_ycaf226 [file supplemental_figure_5_r1_ycaf226.pdf]

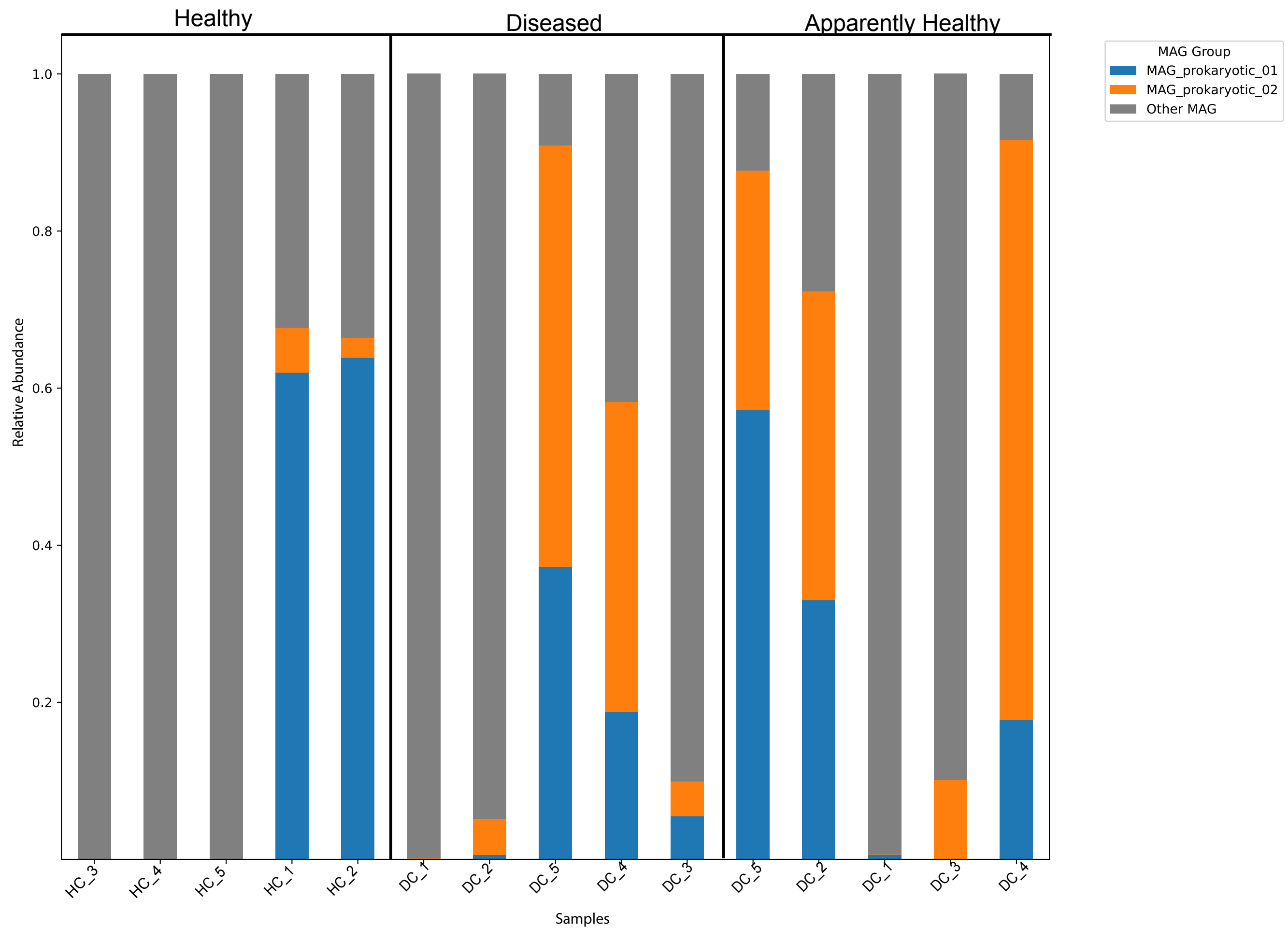

Supplement: Supplemental_Figure_6_ycaf226 [file supplemental_figure_6_ycaf226.pdf]
