# Supplementary material for: Shifts in the microbiome and virome are associated with stony coral tissue loss disease (SCTLD)
Source: ISME Commun. 2025 Nov 27;5(1):ycaf226. doi: 10.1093/ismeco/ycaf226 (PMC12743298; doi:10.1093/ismeco/ycaf226)
Supplement: ISMECOMMUN_Supplemental_Text_R1_ycaf226 [file ismecommun_supplemental_text_r1_ycaf226.docx]

**Supplemental Text: Shifts in the microbiome and virome are associated with stony coral tissue loss disease (SCTLD)**

**Methods**

*Verifying vMAGs and putative host identification*

A common challenge when isolating vMAGs in metagenomic data is the mis-annotation of host contigs as viral. To confirm viral origin and putative hosts, proteins from vMAGs associated with SCTLD were assessed using phylogenetics. DIAMOND blastp (v2.1.10.164; --ultra-sensitive --max-target-seqs 0 --evalue 1e-05) [1] was used to search the vMAG proteins against two databases, nr (2022_07) and IMG/VR (v4.1) [2]. The hits returned by this analysis were too voluminous for phylogenetic analysis (computationally intractable or too large to visualize), necessitating the application of downsampling. For each query protein, hits to the **nr** database were downsampled, retaining only the **top hit for each taxonomic class (i.e., for each taxonomic class in the resulting hits, just the top hit with the lowest *e*-value was retained)**. For hits to the IMG/VR database, only the top 50 hits (based on bitscore) were selected for phylogenetic analysis (due to the absence of reliable taxonomy information for many of the sequences in IMG/VR). For each query, the protein sequences of the hits which remained after downsampling were combined, along with the query protein itself, into a file for downstream analysis. For each of the resulting protein files, MAFFT (v7.520; --auto) [3] was used to generate an alignment and trimAl (v1.5.rev0; --automated1) [4] was used to trim poor quality sequences and positions. Maximum Likelihood (ML) phylogenetic trees were generated for each trimmed alignment using iqtree (v2.3.6; -m TEST -bb 1000) [5]. The placement of the query protein in each tree was assessed relative to all viral and non-viral proteins to verify true viral origin and potentially identify putative hosts. The function of each viral protein was assessed using **GhostKOALA (v3.1.0) [6]**.

**Results and Discussion**

*General metagenome analysis*

Metagenomic short read sequencing produced 249-321 million read pairs per sample, which constituted 74.8-113.5 Gbp of data per sample (**Supplemental Table 8**). As expected, read deduplication, quality filtering, and mapping to the preliminary *D. labyrinthiformis* genome generated from the first round of sequencing significantly reduced the total amount of data for binning, with many samples reducing in size by >90%. This is an expected result because most of the read data will be of host provenance and is removed during the mapping stage. Removal of this data improves both the runtime and accuracy of metagenomic workflows. After read processing, binning, and quality assessment we obtained a total of 264 MAGs which exceeded the minimum MAG quality cutoffs used for each taxonomic domain (**See Supplemental Text**). Of the 264 MAGs, two were eukaryotic, a sponge and a diatom, which had BUSCO completeness scores of 23.9% and 11% (respectively). Owing to their low completeness these eukaryotes were not included in further downstream analysis. A total of 85 MAGs were prokaryotic (pMAGs), 23 were classified as “Complete” (completeness > 90% and contamination < 5%, as assessed by CheckM), 39 were classified as “High-quality” (completeness > 70% and contamination < 10%), and 23 were classified as “Medium-quality” (completeness > 50% and contamination < 10%) (**Supplemental Table 1**). Additionally, we identified 36 plasmid MAGs, with a geNOMAD plasmid score > 0.85 and a false discovery rate (FDR) < 0.05, and 141 viral MAGs (vMAGs), with a geNOMAD score > 0.90 and an FDR < 0.05. Of the 141 vMAGs, 109 were classified as Caudoviricetes, which are widely distributed bacteriophages (**Supplemental Table 2**).

*Virome phylum level analysis*

Phylum-level taxonomic analysis of the virome was performed to assess the overall shifts in the viral community between the different health conditions. In HC samples, Uroviricota was the most abundant phylum (0.5094 ± 0.2083), followed by Nucleocytoviricota (0.2054 ± 0.1410). In AH samples, the most abundant phylum shifts to Nucleocytoviricota (0.4496 ± 0.1002); this shift was large enough to reach statistical significance (*p*-value = 0.035). A decrease of Uroviricota (0.3636 ± 0.0429) was observed in the AH samples, although it was not statistically significant. Lastly in DL samples, the opposite pattern was observed: i.e., Uroviricota was the most abundant (0.4083 ± 0.0432), followed by Nucleocytoviricota (0.3762 ± 0.1176). Log₂TPM fold change values for each of the vMAGs were also examined to capture general trends in the virome (**Supplemental Figure 3**). We observe an increased abundance of 18 vMAGs in AH samples and 33 vMAGs in DL samples (all compared to HC samples), of which 16 were shared. Furthermore, 97 vMAGs have a decreased absence in AH samples and 95 vMAGs in DL samples.

These results show that there is a notable shift in viral community composition across health conditions. Specifically, HC samples were dominated by Uroviricota, particularly Caudoviricetes, which are bacteriophages typically associated with environmental microbiomes. In contrast, AH samples were dominated by Nucleocytoviricota, a phylum of double-stranded DNA viruses. Interestingly, Uroviricota abundance showed a partial resurgence in DL samples, where lesions were already established, and significant tissue loss had occurred. The decline of Uroviricota in AH samples aligns with shifts in the prokaryotic microbiome, their primary host, suggesting a tight coupling between viral and bacterial community dynamics. The subsequent resurgence in DL samples likely results from bacteriophage infection of newly colonizing bacteria. Moreover, the high diversity of vMAGs in healthy colonies makes it challenging to attribute a biologically significant contribution to any single vMAG. However, general virome trends can be utilized, and show that a majority of vMAGs decrease in relative abundance in samples from diseased colonies (DC).

*Prokaryote abundance results*

It is important to note that in one infected colony, samples DC_1 (AH) and DC_1 (DL), we observe low Leptospirales and Gammaproteobacteria abundance. All other infected colonies showed an increased prevalence of these pMAGs, suggesting an alternative opportunistic pathogen was present in samples N35 and N33. This idea is corroborated by other SCTLD studies that have identified different putatively pathogenic bacteria associated with infection. These other studies have found Rhodobacterales, Rhizobiales*,* and *Vibrio* spp. [7, 8] to be enriched in diseased corals, however, our analysis did not identify these bacteria, suggesting that the secondary infection agent may differ across coral species and locations.

*Microbiome profile shifts under infection*

An initial relative abundance analysis of the microbiome was performed at the “class” taxonomic level (**Supplemental Table 1**). In HC samples, Alphaproteobacteria was the most abundant class (0.3918 ± 0.2209), followed by Gammaproteobacteria (0.1461 ± 0.2585) (**Figure 2A**). In AH samples, Gammaproteobacteria was the most abundant (0.3432 *±* 0.3603) and Leptospirales was the second most abundant (0.3047 ± 0.2873), and Alphaproteobacteria was the third (0.0159 *±* 0. 1945). In DL samples, Gammaproteobacteria was the most abundant (0.4207 ± 0.2397), followed by Alphaproteobacteria (0.1538 ± 0.2317) and *Leptospirae* (0.0453 ± 0.2444). The increase in the class *Leptospirae* was statistically significant in both AH and DL samples compared to the HC samples (*p*-value = 0.035 for both).

We assessed **differential abundance** of individual pMAGs using DESeq2 (|log₂FC| > 2.0 and an adjusted *p*-value < 0.05). Out of the 85 pMAGs, three showed a significant increase in infected colonies, in both AH and DL samples. In addition, seven pMAGs showed significantly decreased abundance in AH samples, and eight in DL samples. Only one pMAG was not decreased in AH but decreased in DL. To evaluate broader patterns beyond statistical significance, we calculated log₂TPM-based fold changes. Although this analysis did not assess statistical significance, it revealed that 16 pMAGs had increased abundance in infected colonies, whereas 69 pMAGs showed decreased abundance, suggesting a general trend of microbial decline across infected colonies (in either HC_vs_DL or HC_vs_AH; **Figure 2B**). To interpret biological significance of these shifts, we looked at the relative abundance of each of the statistically significant pMAGs. In infected colonies, three pMAGs had an increased abundance: MAG_prokaryotic_02, MAG_prokaryotic_01, and MAG_prokaryotic_45. In HC samples, we observe that the relative abundance of these MAGs was 0.000127 ± 0.000117, 0.000003 ± 0.000003, and 0.000264 ± 0.000103, respectively. In AH samples, an increase in relative abundance (compared to HC samples) was observed, 0.304739 ± 0.287329, 0.177501 ± 0.240849, and 0.009025 ± 0.129915. In the DL samples, the relative abundance was higher than in the HC samples but lower than the AH samples, 0.045344 ± 0.244371, 0.055040 ± 0.158030, and 0.015674 ± 0.062248. pMAGs that had a decreased abundance, i.e., abundant in healthy but less abundant in infected colonies, were more challenging to assess due to the high microbiome richness and diversity in the HC samples. That is, pMAGs in healthy colonies tended to be specific to a single colony and not broadly shared across other colonies, confounding statistical analysis. Given these considerations, pMAGs were considered of biological interest if they were differentially abundant and had a median relative abundance > 0.1 in at least one health condition. As a result, MAG_prokaryotic_02 and MAG_prokaryotic_01 were selected for downstream analyses.

*vMAG phylogenetic analysis*

Of the 41 proteins from the five SCTLD-associated vMAGs, we were able to generate phylogenetic trees for 25 (**Supplemental File 1**). vMAG058 had no genes with sufficient hits to nr or IMGV for phylogenetic reconstruction and thus could not be assessed by this analysis. For vMAG001 (geNOMAD taxonomy: Adintoviridae), 11/15 genes had phylogenies constructed, all of which contained hydra or coral sequences intermixed with known viral and bacterial sequences (**Supplemental Table 6**). Furthermore, 10 of the trees contained hydra adintovirus sequences and 2 contained *Stylophora* adintovirus sequences, supporting vMAG001 as putatively host infecting. For vMAG043 (geNOMAD taxonomy: Imitevirales), 3/7 genes had phylogenies constructed. Two of the constructed phylogenies contained the coral virus *Rhodactis* coral adintovirus and the coral *Acropora millepora*. For vMAG055 (geNOMAD taxonomy: Megaviricetes), 5/9 genes had phylogenies constructed. None of the phylogenies contained any corals, however 2 trees contained coral endosymbiont sequences from *Symbiodinium microadriaticum* and *Symbiodinium natans*. Lastly, for vMAG060 (geNOMAD taxonomy: Caudoviricites), 7/8 genes had phylogenies constructed. All seven trees contained either corals or hydras sequences, and only one gene contained *Corynactis* coral adintovirus, suggesting that vMAG060 may be a putative host infecting virus. However, across all vMAGs discussed, the majority of trees also included other bacteria, making it difficult to unambiguously identify the host.

**References**

1. Buchfink B, Xie C, Huson DH. Fast and sensitive protein alignment using DIAMOND. *Nat Methods* 2015;**12**:59–60. https://doi.org/10.1038/nmeth.3176

2. Camargo AP et al. IMG/VR v4: an expanded database of uncultivated virus genomes within a framework of extensive functional, taxonomic, and ecological metadata. *Nucleic Acids Research* 2023;**51**:D733–D743. https://doi.org/10.1093/nar/gkac1037

3. Katoh K. MAFFT: a novel method for rapid multiple sequence alignment based on fast Fourier transform. *Nucleic Acids Research* 2002;**30**:3059–3066. https://doi.org/10.1093/nar/gkf436

4. Capella-Gutiérrez S, Silla-Martínez JM, Gabaldón T. trimAl: a tool for automated alignment trimming in large-scale phylogenetic analyses. *Bioinformatics* 2009;**25**:1972–1973. https://doi.org/10.1093/bioinformatics/btp348

5. Minh BQ et al. IQ-TREE 2: New Models and Efficient Methods for Phylogenetic Inference in the Genomic Era. *Molecular Biology and Evolution* 2020;**37**:1530–1534. https://doi.org/10.1093/molbev/msaa015

6. Kanehisa M, Sato Y, Morishima K. BlastKOALA and GhostKOALA: KEGG Tools for Functional Characterization of Genome and Metagenome Sequences. *J Mol Biol* 2016;**428**:726–731. https://doi.org/10.1016/j.jmb.2015.11.006

7. Rosales SM et al. Rhodobacterales and Rhizobiales Are Associated With Stony Coral Tissue Loss Disease and Its Suspected Sources of Transmission. *Front Microbiol* 2020;**11**:681. https://doi.org/10.3389/fmicb.2020.00681

8. Ushijima B et al. Disease Diagnostics and Potential Coinfections by Vibrio coralliilyticus During an Ongoing Coral Disease Outbreak in Florida. *Front Microbiol* 2020;**11**:569354. https://doi.org/10.3389/fmicb.2020.569354
